# Engineering Polyketide Stereocenters with Ketoreductase Domain Exchanges

**DOI:** 10.1021/jacs.5c06736

**Published:** 2025-11-04

**Authors:** Leah S. Keiser, Panarai Primrose Gatenil, Yolanda Zhu, Kai Deng, Lucas Waldburger, Jennifer W. Gin, Yan Chen, Edward E. K. Baidoo, Christopher J. Petzold, Nathan Lanclos, Trent R. Northen, Elias Englund, Jay D. Keasling

**Affiliations:** † 124489 Joint BioEnergy Institute, Emeryville, California 94608, United States; ‡ Department of Chemical and Biomolecular Engineering, University of California, Berkeley, California 94720, United States; § Biological Systems & Engineering Division, 1666 Lawrence Berkeley National Laboratory, Berkeley, California 94720, United States; ∥ Department of Bioengineering, University of California, Berkeley, California 94720, United States; ⊥ Department of Biomaterials and Biomanufacturing, Sandia National Laboratories, Livermore, California 94550, United States; # Environmental Genomics and Systems Biology Division, 1666 Lawrence Berkeley National Laboratory, Berkeley, California 94720, United States; ∇ School of Engineering Sciences in Chemistry, Biotechnology and Health, Science for Life Laboratory, KTH − Royal Institute of Technology, 106 91 Stockholm, Sweden; ○ California Institute for Quantitative Biosciences (QB3), University of California, Berkeley, California 94720, United States; ◆ The Novo Nordisk Foundation Center for Biosustainability, Technical University of Denmark, Lyngby 2800, Denmark

## Abstract

Polyketide synthases (PKSs) are versatile biosynthetic megasynthases capable of producing a diverse range of natural products with many applications, including in pharmaceuticals. The stereochemical precision of PKSs makes them a powerful tool for engineering tailored, unnatural polyketides; however, modifying the stereocenters of a PKS product while maintaining production levels remains a significant challenge. In this study, we systematically tested and evaluated strategies for ketoreductase (KR) domain exchanges, the domain responsible for setting stereocenters of polyketide products. After first optimizing the method for KR exchanges, we then performed 44 KR domain exchanges on three different PKSs to obtain high production of all four stereoisomers in vivo. By testing both one- and two-module PKS systems, we investigated how downstream modules process intermediates with altered stereochemistry and found that the configuration of the α-substituents was critical for gatekeeping by the ketosynthase (KS). To overcome this constraint, we investigated two different strategies for altering the KS domain, including introducing targeted mutations in the downstream KS, and exploring boundaries in exchanging the entire functional unit from the donor PKS. Both strategies successfully modified the KS stereocontrol with distinct trade-offs; the functional unit exchange resulted in higher titer improvements, though it was more likely to break the entire PKS. This study demonstrates a comprehensive approach to successfully engineering all four stereochemical configurations in multiple PKS systems, advancing our understanding of and ability to rationally modify polyketide stereochemistry through multiple engineering strategies.

## Graphical abstract

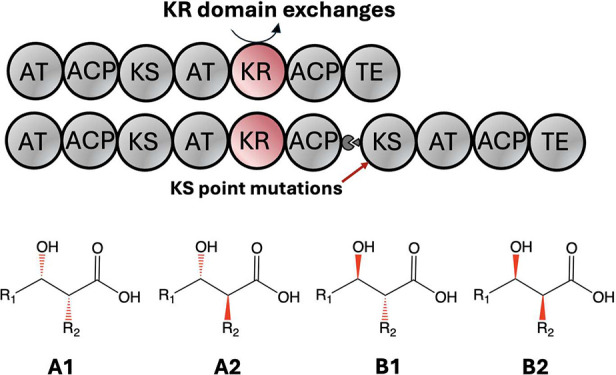

## Introduction

Natural polyketides have been utilized in a wide range of pharmaceuticals, including antivirals, antiparasitics, antibiotics, antifungals, antitumor agents, and anticancer drugs.^1–9^ This versatile class of secondary metabolites is rich in asymmetric centers, which is important as even slight modifications in stereochemistry can significantly influence biological activity.^10–12^ An example of the role of stereochemistry in polyketide activity is the C8 epimer of erythromycin B, a macrolide antibiotic. Inverting this single stereocenter reduced the antibiotic activity of the macrolide to just 3% of the original compound.^13^ While the traditional chemical synthesis of pure stereoisomers is often challenging, biologically based type 1 polyketide synthases (PKSs) naturally provide high stereochemical precision.

PKSs are multimodular, megasynthases that play a critical role in the synthesis of complex and biologically active polyketides. These enzymes function in an assembly line manner, enabling the retrobiosynthesis of engineered unnatural products from simple precursors.^14,15^ Their modular architecture makes them of interest in designing tailor-made products for medical and industrial applications, as polyketide design can be achieved solely through host DNA modification using traditional synthetic biology techniques.^16^ However, despite recent advances in PKS engineering and chimeric PKS design, reliably and predictably controlling product stereochemistry while maintaining the titers associated with the native stereochemistry remains a significant hurdle.

A PKS consists of multiple domains organized into modules. Each module contributes a single ketide unit to the growing chain by mediating its extension and modification. The core domains in a PKS module include the ketosynthase (KS), which catalyzes the Claisen-condensation reaction; the acyltransferase (AT), which selects the acyl-CoA loading or extender unit; and the acyl carrier protein (ACP), which tethers the intermediate via a thioester linkage. The chain is typically released through cleavage by a thioesterase (TE) domain. Additional structural modifications are introduced by the reductive loop, composed of the ketoreductase (KR), dehydratase (DH), and enoylreductase (ER) domains, with KR domains being the primary focus of this study.

The KR domain of a PKS sets the stereocenters of the polyketide products by catalyzing the epimerization of the α-substituent and the NADPH-dependent reduction of the β-hydroxy group on the nascent chain.^17,18^ Based on the stereochemical outcomes of the α- and β-groups, KR domains are classified into four types, specifically A1, A2, B1, and B2, each associated with distinct key active site residues (Figure 1).^19,20^ Additional types of KR domains are C-type KRs that lack reductive ability.

**Figure 1:**
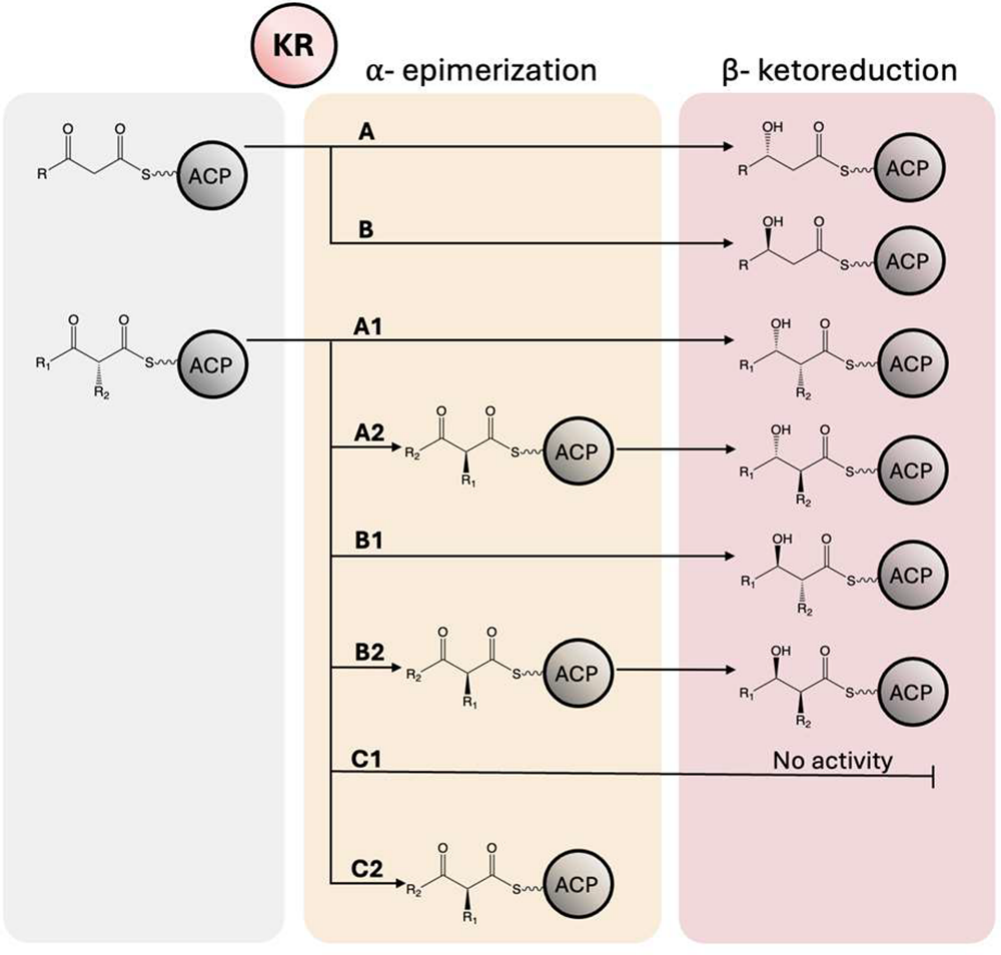
KR-types and their stereochemical outcomes. The KR domain in a PKS is responsible for both epimerization and reduction of the ketide intermediate, resulting in eight possible chemical outcomes depending on the extension substrate and the KR-type.

Previous efforts to alter polyketide stereochemistry have broadly followed two different strategies. The first uses point mutations to alter the active site residues associated with one specific KR type to that of another type. This strategy has seen success when used on individual isolated KR domains purified and assayed in vitro, but has failed to change the stereocenters when the KR domains are attached to whole PKS modules.^21–24^ The other strategy employs domain exchanges, replacing the native KR domain entirely with a KR domain of a different type.^25–30^ Since this approach replaces an entire domain, it introduces the added complexity of potential protein–protein interface disruptions between the inserted domain and the rest of the PKS, creating the need to select domain boundaries with minimal interference.

Previously, the A1-type KR in the second module of the erythromycin PKS, 6-deoxyerythronolide B synthase (DEBSM2) has been successfully exchanged with α-epimerizing A2-type KRs; however, substituting B-type KRs has been more difficult.^26–30^ While these previous studies in DEBS have detected the B1-type product in various amounts, exchanges with B2-type KRs abolished KR activity in most cases.^27–30^ Experiments on the A2-type KR of module 1 of the lipomycin PKS (Lip1-TE) have seen similar results, where KR domain exchange with A1-types retained their activity while none of the tested B1- or B2-type exchanges produced a reduced product.^28^ In the most effective B2-type stereochemistry exchange to date, Massicard et al. produced the correct stereoisomer by performing an AT-KR-ACP tridomain exchange into DEBSM2, but with a significant drop in product titer.^29^ Although these previous studies have demonstrated that KR domain exchange is possible in some cases, the most common outcome is either a significant loss in activity of the chimeric PKS or the complete abolishment of it. The reason behind the success of some exchanges and the failure of most is poorly understood, demonstrating the need for a more systematic study to uncover the underlying mechanism and improve consistency.

In this study, we address these challenges through a comprehensive and systematic investigation of KR domain exchanges across multiple PKS systems. We investigated factors critical to successful domain exchanges, including the optimal boundaries for KR domain exchanges, the role of dimerization elements, and the stereochemical gatekeeping mechanism of downstream KS domains. Using our refined approach, we successfully achieved all four possible stereochemical configurations (A1, A2, B1, and B2) in all three systems. These insights provide a robust foundation for more predictable engineering of polyketide stereochemistry, addressing a critical barrier in the design of novel, biologically active compounds with potential pharmaceutical applications.

## Results

To determine the optimal strategy for exchanging KR domains, we utilized Lip1-TE which consists of the loading and first extension modules of the lipomycin PKS fused to the DEBS TE (Lip1-TE) as previously described (Figure 2a).^31,32^ We first assessed whether replacing only the KR domain or incorporating additional flanking domains (AT and/or ACP) would better preserve the native protein–protein interactions within the chimeric PKS. Three donor KR domains were selected for this investigation, each representing a different, non-native stereochemical type: an A1-type from the aculeximycin PKS, a B1-type from the bafilomycin PKS, and a B2-type from the erythromycin PKS. Those KR donors were selected based on their native substrate similarity to Lip1-TE, as each was derived from the first extension module of their respective PKSs and process methylmalonyl-CoA extender units. For each donor PKS, we constructed four variants of Lip1-TE with different exchange strategies, with either the KR alone, AT-KR, KR-ACP, or AT-KR-ACP replacement. The unmodified Lip1-TE and a catalytically inactive KR variant Lip1-TE (KR*) were used as controls.^31^ All engineered PKSs were expressed in *Streptomyces albus* J1074, and we quantified the production of the reduced 3-hydroxyacid product, the unreduced ketone product, and the stereochemistry of the α-methyl and β-hydroxyl groups of the reduced 3-hydroxyacid through GC-MS and LC-MS analysis with comparison against authentic standards. Domain boundaries are summarized in Figure S1, and standard curves for quantification are shown in Figure S2.

**Figure 2:**
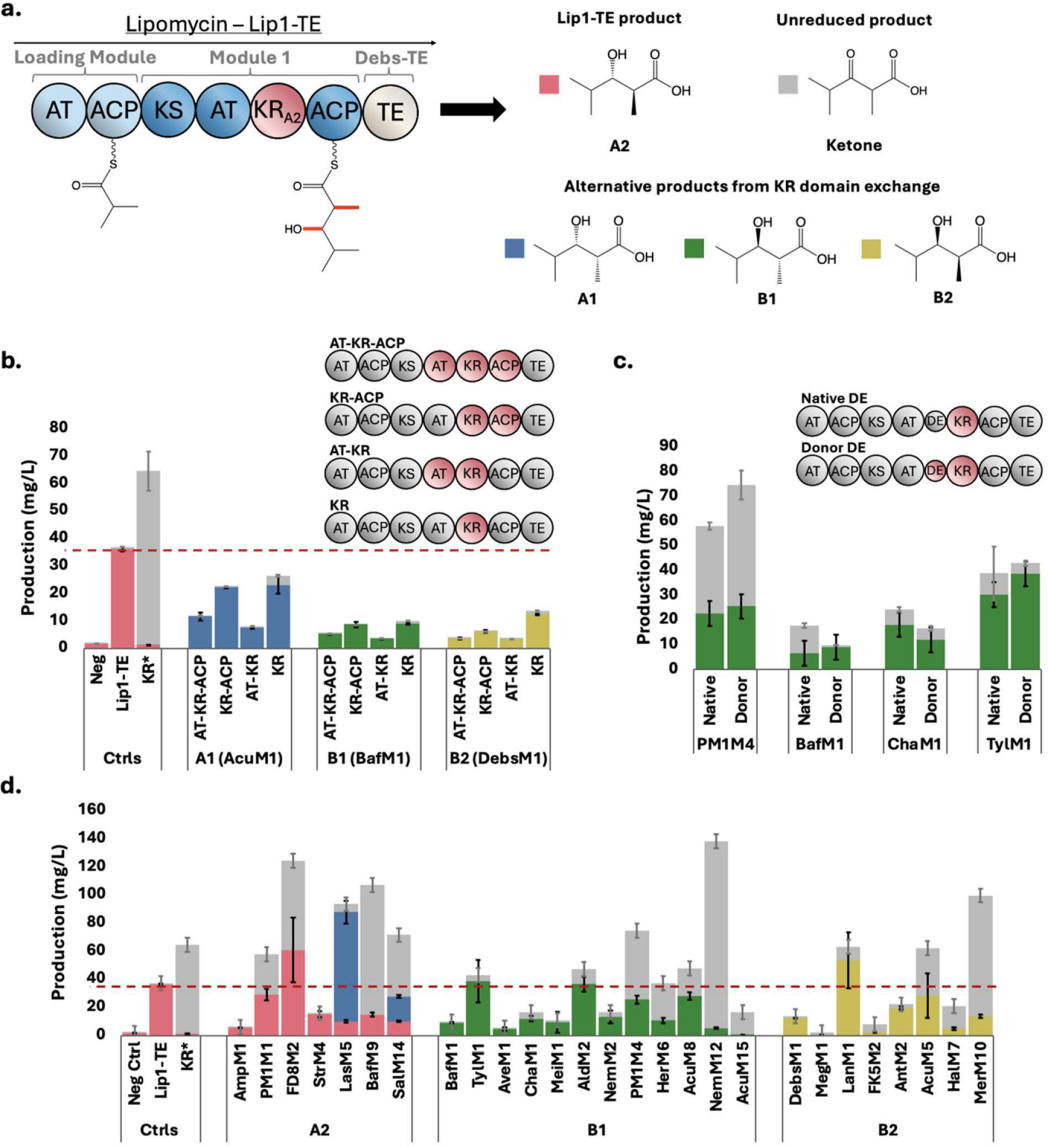
Polyketide production by *S. albus* harboring the Lip1-TE PKS with various KR domain exchanges. (a) Lip1-TE PKS loads isobutyl-CoA and extends with methylmalonyl-CoA, producing either the 3-hydroxyacid product or the unreduced ketone product. All figures use the same colors for each KR-type stereoisomer and the unreduced ketone byproduct. (b) KR domain was either exchanged alone, with the AT or ACP domain, or with all three domains. The controls are *S. albus* J1074 harboring mCherry as a negative control, Lip1-TE with the native KR, and Lip1-TE (KR*), with the KR inactivated for ketone-only production. (c) DE domain was examined by exchanging the KR domain either with the native DE or the donor DE, with four different B1-type KR donors. (d) KR domain was exchanged using the donor DE when available to make the A2-, B1-, and B2- type products in the natively A2-type Lip1-TE. Data are presented as mean values of three biological replicates, and error bars show standard deviation.

With all three donor PKSs, we observed functional production of the reduced 3-hydroxyacid with stereochemistry corresponding to the introduced KR domains (Figure 2b). While titers were lower than that of the unmodified Lip1-TE (36.1 mg/L), almost all of the products were in the reduced form, confirming functional KR activities. Among the tested strategies, exchanging the KR domain alone yielded the highest 3-hydroxyacid titers, followed by a KR-ACP exchange. The least effective strategy involved inclusion of the AT domain, regardless of whether the ACP domain was also included. This result is somewhat unexpected, as the revised domain boundaries place the natural module junctions between the KS and AT domains.^33^ However, in the context of PKS engineering, exchanging multiple domains might be more structurally destabilizing to Lip1-TE than replacing only a single domain. Based on this limited data set, we concluded that a KR-only exchange was the strategy with the highest success rate and used it for all subsequent experiments.

Next, we tested the effect of dimerization elements (DEs) on KR domain exchanges. DEs are a ∼55-amino acid region between the AT and KR domains in about half of KR-containing PKSs; they play a role in stabilizing the dimeric PKS structure.^34^ The DE has been reported to influence the success of KR domain exchanges, particularly when introducing a KR associated with a DE into a system that lacks one.^28^ To systematically investigate the role of the DE, we selected four B1-type KR domains: two with native DEs (BafM4 and PM1M4) and two without (TylM1 and ChaM1), and introduced them into Lip1-TE. We tested two variants from each donor KR, one where the native Lip1-TE DE was maintained and one where it was exchanged with the donor DE when present or removed entirely for the cases of KR donors without a DE (Table S1). Our results show no clear impact of DE presence or absence on KR performance, with similar titers being produced by either form (Figure 2c). While previous studies demonstrated that DE choice can impact KR domain exchange success, we observed no significant difference with these B-type donors.^28^ Therefore, we decided to retain the donor DE when present in future exchanges to preserve the natural domain architecture.

Using the optimized KR domain exchange strategy, we performed a survey of KR exchanges in Lip1-TE, replacing the native A2-type KR domain. As we have previously shown that we can successfully implement A1-type KR domain exchanges into Lip1-TE in vitro, we focused on the A2, B1, and B2 exchanges. We used the web-based toolkit ClusterCad to identify 7 A2-type KRs, 12 B1-type KRs and 8 B2-type KR domains from PKS modules which possess an AT domain that naturally uses methylmalonyl-CoA extender units.^28,35^ These KR domains were cloned into Lip1-TE, expressed in *S. albus*, and production was then measured. Of the 27 tested KR exchanges, one A2-type (60.9 mg/L FD8M2), two B1-type (38.7 mg/L TylM1, 36.5 mg/L AldM2) and one B2-type (53.3 mg/L LanM1) KR domain exchanges produced titers similar to or exceeding that of the unmodified Lip1-TE (Figure 2d). While some exchanges nearly abolished Lip1-TE activity, most retained at least 25% of wild-type production levels of the reduced product (6 out of 7 A2-type KRs, 9 out of 12 B1-type KRs, and 5 out of 9 B2-type KRs). Notably, some KR exchanges produced high titers of the ketone product despite low production of the reduced form. For example, the NemM12 KR produced 133.1 mg/L of the unreduced ketone product, more than twice the amount produced by the catalytically inactive KR* Lip1-TE control (63.1 mg/L). Since the KR* variant has been used to produce over 1 g/L short chain ketones in *S. albus* under optimized fermentation conditions, the NemM12 variant could be a more efficient ketone-producing PKS.^31^

Stereochemistry of all products were measured using GC-MS, and the retention times were compared against enantiomerically pure standards (Figure S3, Table S2). The analysis showed that most KR domain exchanges produced the expected configuration, except for two A2-type KR domains, LasM5 and SalM14, which produced 89 and 64% of their product in the unepimerized α-methyl A1 form, respectively. Notably, LasM5 was the highest overall producer of reduced products, yielding 87.7 mg/L of combined A1 and A2 products. The failure of LasM5 to uniformly produce the expected stereoisomer could be because the epimerization step is rate limiting and therefore the product is reduced before being successfully epimerized, which seems to be supported by the high titers produced by LasM5. However, this explanation does not hold for SalM14, which produced only a small amount of reduced product. Another factor could be the preference of DEBS-TE for A1- and B2-type stereoisomers over A2- and B1-forms.^36^ The preference for the unepimerized A1-form could mean that the TE is prematurely hydrolyzing the intermediate, even though the α-methyl epimerization occurs prior to β-keto reduction.^37^

We included A2-type KR domain exchanges to test the effect of introducing a foreign domain into a PKS without altering the final product, allowing us to distinguish between stereochemical modification of the intermediate and the effect of exchanging one domain for another and potentially interfering with the protein–protein interactions. However, despite catalyzing the native reaction, A2-type KR exchanges were no more successful than the nonnative B1- and B2-type exchanges. Most A2 KR domain exchanges led to a decreased amount of the reduced product compared to the wild-type KR, and/or a substantial accumulation of the unreduced ketone product. This indicates that the reduced activity of the KR domain exchanges, where overall only 4 out of 27 exchanges had equal production to Lip1-TE, is not primarily through gatekeeping by the Lip1-TE domains, but due to factors such as lower activity of the donor KR, less favorable ACP - KR interactions, or by protein destabilization from the introduced domain.^38^ Since Lip1-TE is a single-extension PKS, gatekeeping is unlikely to be the primary limiting factor, as only the TE domain acts on the nascent polyketide chain after the KR.

From our generated data set of 27 KR exchanges in Lip1-TE, we investigated whether chemical similarity, or chemosimilarity, between the substrate of a donor KR and the native Lip1-TE substrate could predict KR domain exchange success. A prior study in reductive loop exchanges (KR, DH and ER domains) suggested that chemosimilarity might be a key determinant of success for multidomain exchanges.^39^ To test the role of chemosimilarity with KR domain exchanges, we calculated the chemosimilarity values for all donor KR domains relative to the native Lip1-TE (Figure S4a,b). The Spearman correlation analysis revealed only modest relationships between chemosimilarity and product titers. While a weak positive correlation was found with 3-hydroxyacid production (ρ = 0.23), there was a stronger negative correlation with ketone production (ρ = −0.57), suggesting that KR domains with a higher chemosimilarity to the native Lip1-TE KR were less likely to produce the unreduced ketone byproduct. This indicates that while chemosimilarity may not strongly predict overall productivity, it might relate to the completeness of reduction.

Among the top performing exchanges that produced 3-hydroxyacids at titers comparable to the native Lip1-TE were FD8M2, TylM1, AldM2, and LanM1, with chemosimilarity ranging from moderate (0.484 in TylM1) to relatively high (0.6525 in FD8M2). However, the trend was inconsistent across the data set, as several KR domains with high chemosimilarity values produced low titers, while some with lower values performed reasonably well. These results suggest that while chemosimilarity may contribute to domain exchange success, it cannot serve as the sole predictor of performance. The analysis of the total overall production of 3-hydroxyacid and ketones further supports this conclusion, as even domains with similar chemosimilarity values showed substantially different production profiles. Additional factors, likely including protein–protein interactions between the introduced KR domain and the PKS acceptor, appear to play critical roles in determining success of KR domain exchanges.

To investigate whether the change in titers associated with PKS engineering were reflective of intrinsic catalytic properties or simply changes in protein content from strain to strain, we performed targeted proteomics on Lip1-TE KR domain exchanged strains to investigate possible relationships between the amount of PKS and the measured titers (Figure S5). The Lip1-TE KR domain exchanges in *S. albus* showed a moderate positive correlation between protein abundance and production (Pearson *r*_*p*_ = 0.49, *p* = 0.007 and Spearman *r*_*s*_ = 0.55, *p* = 0.002). However, protein abundance only explained about 24% of the measured variance (*r* ^2^ = 0.24), demonstrating that differences in titers are multifactorial. While some of the measured differences in titers could be explained by changes in the expression and solubility of the proteins, this analysis shows that at least some of the differences in measured titers of the 3-hydroxyacids are likely related to the altered intrinsic activity of the engineered variants.

Specifically, LasM5 had comparatively low protein expression, but one of the highest titers, giving it the highest production per unit protein, near the WT Lip1-TE (Figure S5). Conversely, LanM1, a high performer on titers alone, had one of the highest levels of protein expression, which shows that the change is likely largely due to the difference in protein expression and not because of catalytic changes. Some domain exchanges, like that with BafM9 or StrM4, had higher protein expression and lower titers, which demonstrates that high titers are certainly not solely correlated with higher protein expression and solubility. While protein content explains nearly a quarter of the variance, catalytic properties and domain compatibility likely remain the dominant factors in determining the effectiveness of the KR domain exchanges.

While Lip1-TE is an effective model for single-extension PKSs, we extended our investigation to two-module systems to assess the tolerance of a downstream module for intermediates with altered stereochemistry, something that is important for understanding how KR exchanges would function in full size PKSs. For this, we employed the chimeric pikromycin PKSs developed by Miyazawa et al., designated Pik127 and Pik167, that make triketide lactones (Figure 3a). Pik127 contains a B2-type KR in the first extension module, and Pik167 contains an A1-type KR in the first extension module.^40^ This arrangement allowed us to explore how the KS domain downstream of the first module gatekeeps against the stereochemistry from its upstream domains. Although the KS domain is known to act as the primary gatekeeper for PKS chain elongation, the extent to which a KS is able to accept a substrate based on the stereochemistry remains unclear.^33,41,42^

**Figure 3:**
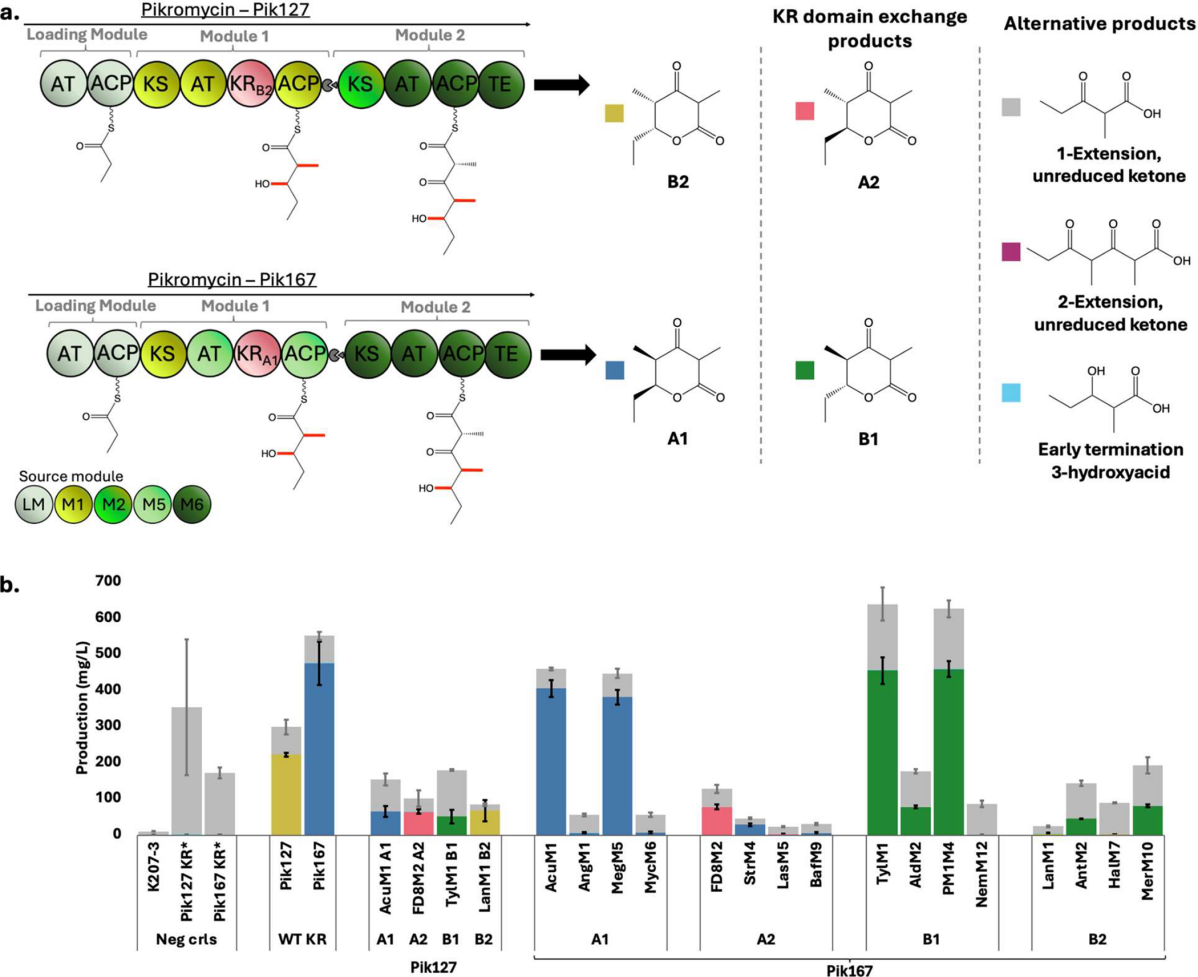
In vivo production data of the chimeric, two-module PKS systems Pik127 and Pik167 expressed in *E. coli* K207-3. (a) Both Pik127 and Pik167 are two-polypeptide PKSs joined by N- and C-terminal docking domains, where the colors indicate which module each pikromycin domain is sourced from. The five products made include the A1-, A2-, B1-, and B2-type variants of the triketide lactone, along with the corresponding unreduced ketone products and the 3-hydroxyacid product made when the PKS fails to extend the intermediate. Unlike the one-extension ketone intermediate, the two-extension ketone intermediate was not detected in any strains. (b) KR domain exchanges of all four types were performed in both Pik127 and Pik167. The controls were either the plasmid containing either the first or second part of the PKS, the KR* variant for ketone production, and the PKS with the native, wild-type KR. One KR of each type was tested in Pik127, and four of each type were tested in Pik167. Data are presented as mean values of three biological replicates, and error bars show standard deviation.

Using the same strategy and junctions applied to Lip1-TE, we selected four KR domain donors to make all four stereochemical configurations in Pik127 and 16 KR donors, four of each type, in Pik167. Based on previous studies reporting difficulty in exchanging KR domains from the A1-type stereochemistry and the higher overall production levels reported in Pik167, we opted to expand to a more comprehensive panel of 16 donor KR domains to better investigate more complex stereochemical behavior. The donor KRs were selected based on their performance in Lip1-TE, and an additional three A1-type KRs were selected for exchange into Pik167, each derived from a module with a methylmalonyl-CoA specific AT-domain. To create KR deficient control strains, we mapped the KR mutation for abolishing reduction in Lip1-TE to both chimeric pikromycin PKS systems and mutated the catalytic tyrosine to a phenylalanine (Figure S6). All KR domain exchanged variants of Pik127 and Pik167 were expressed in *E. coli* K207–3, which has been previously optimized for pikromycin production, and to test whether chimeric PKSs created using our optimized domain strategy can work across multiple host systems. The products were again quantified against authentic standards, as shown in Figure S2. The stereochemistry was measured by GC-MS, and the retention times were compared to enantiomerically pure triketide lactone standards (Figure S7, Table S3).

The results from the KR exchange in Pik127 showed successful production of all four isomers but at reduced titers relative to the unmodified enzyme (223 mg/L, Figure 3b). All four KR exchanges made similar amounts of reduced products (∼51 to 68 mg/L), indicating that Pik127 is promiscuous for products with different stereocenters. This result stands in contrast to Pik167 where the KR-type had a large impact on activity. Several KR domain exchanges with A1- and B1-type KR domains achieved high titers, especially with the A1-type AcuM1 KR (406.2 mg/L) and the B1-type PM1M4 KR (459.8 mg/L), though both fell just short of the wild type Pik167 KR (476.1 mg/L). However, for the A2- and B2-type KRs that epimerize the α-methyl, only the A2-type FD8M2 made the correct isomer and in high titer (77.6 mg/L). Two each of the A2- and B2-type KR domains were unable to epimerize the intermediates, resulting in the A1- and B1-type products, respectively. The native A1-type KR in Pik167 lacks epimerization ability, which could explain why the success rate and titers were much lower in cases where the donor KR domain requires epimerization. However, Pik127 does have epimerization activity, and we did not see selectivity against the nonepimerized α-methyl group for Pik127 with the tested KR exchanges, highlighting an important difference between the two PKS systems.

Similar to our analysis of Lip1-TE, we investigated whether chemosimilarity could predict successful KR domain exchanges in Pik167 (Figure S4c). The Spearman correlation revealed a slightly different trend compared to Lip1-TE, notably where there is almost no correlation between chemosimilarity and ketone production (ρ = −0.14) (Figure S 4d). However, we observed a moderate positive correlation between chemosimilarity and triketide lactone production (ρ = 0.43), suggesting that in Pik167, higher chemosimilarity may somewhat favor the production of the desired reduced product, though the relationship is not strong enough to serve as a reliable predictor alone. Among the most successful exchanges in Pik167, the A1-type AcuM1 KR and B1-type PM1M4 KR domains showed relatively moderate to high chemosimilarity values. The A2-type FD8M2 KR, which successfully produced the epimerized product, also demonstrated higher chemosimilarity to the native KR. However, several other KR domains with similar chemosimilarity values performed poorly, reinforcing that additional factors significantly influence exchange success.

The results from KR exchanges in Pik167 show that KR-types that have α-methyl epimerization activity had lower success rates than those without. Unlike the single-extension PKS Lip1-TE, Pik167 has two extension modules, with the second module needing sufficient promiscuity to accept the altered intermediate after KR reduction in the first module. Since KS domains are known to act as gatekeepers for polyketide intermediates, we hypothesize that the KS domain in the second extension module of Pik167 preferentially selects intermediates with an α-methyl in the S-configuration.^41^ To test this hypothesis and attempt to enhance the downstream KS’s activity against nonnative stereocenters, we took two complementary approaches. The first involved investigating specific KS residues that could be suitable for point mutations to increase promiscuity toward altered intermediates, and the second involved exchanging larger domain blocks by including the downstream KS domain along with the KR domain. The KS point mutations have the benefit of maintaining the native protein architecture and limiting potential destabilization of the Pik167 protein–protein interactions. However, point mutations tend to offer limited improvements, though this approach in Pik167 KR domain exchanges could reveal more about the specific KS residues involved in gatekeeping against KR stereochemistry.^21^ In contrast, given the suspected evolutionary formation of Type I PKSs through recombination of functional domain units, exchanging the entire AT-KR-ACP-KS unit from the donor PKS could better preserve the natural compatibility between coevolved domains and could potentially offer more substantial titer increases.^33^

To select the residues for point mutations, we referred to a previous study that used bioinformatics to investigate the substrate tunnel residues of several hundred KS domains for sequence fingerprints related to their native substrates.^43^ From this analysis, the authors showed that of the 32 substrate tunnel residues, specific residues were associated with the stereochemistry of the β-hydroxy and α-methyl positions and are therefore associated with an upstream KR type. Based on this work, we selected four KS mutations to apply to Pik167: W236A, L234T, L234M, and L234A. The locations of these residues are indicated on the KS structure shown in Figure 4b.

**Figure 4:**
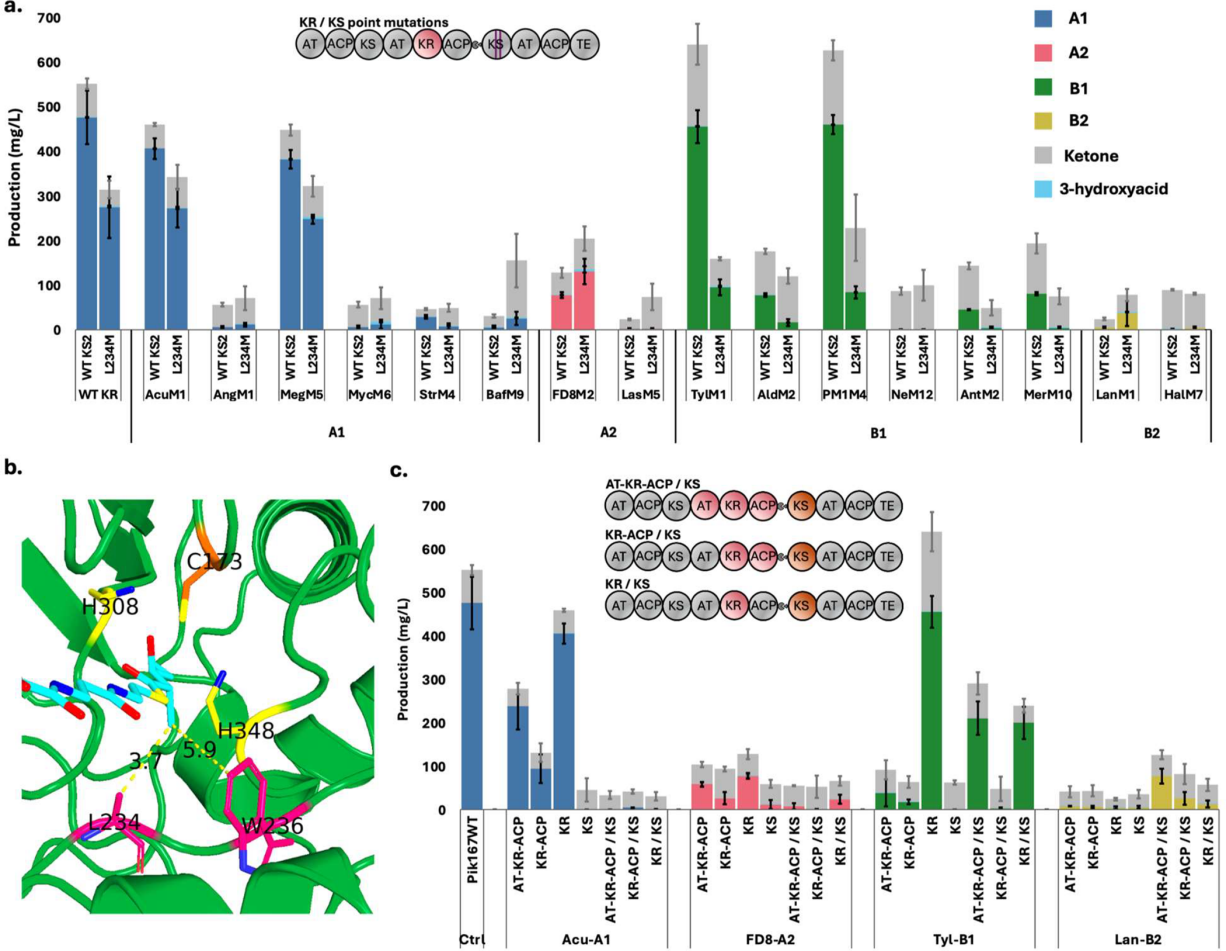
Investigating downstream KS gatekeeping by Pik167 expressed in vivo in *E. coli* K207-3. (a) For one of each kind of KR domain types that were higher producers and exchanged into Pik127, either the AT-KR-ACP, KR-ACP, or KR regions from the donor PKS were exchanged into the first Pik167 plasmid, and they were paired with either the native Pik167 KS or the donor KS where indicated. (b) Predicted structure of the second KS domain in Pik167 is shown, with the catalytic triad (C173, H308, and H348) and the two substrate residues mutated (L234 to L234A/T/M and W236 to W236A) are shown. (c) Effect of the L234 M mutation on triketide lactone production of Pik167 compared with the wild-type KS sequence. Each KR domain exchange in Pik167 was paired with a downstream KS mutation, and the data from the L234 M mutation shown here is grouped by the stereochemistry of the measured products. All other KS mutation variants are shown in Figure S8. Data are presented as mean values of three biological replicates, error bars show standard deviation.

The four selected mutations were performed on the second KS of Pik167 and combined with all 16 KR exchanges, with unmodified Pik167 as control, for a total of 68 combinations tested. The L234 M mutation, which is associated with A2-type KRs, improved product titers of FD8M2. Titers went from 77.6 to 131.0 mg/L, a 69% increase (Figure 4a). When the same mutation was combined with LanM1, a B2-type KR, titers improved 8.1 fold from 4.5 mg/L with the wild-type KS to 36.6 mg/L in the L234 M mutant. These increases in titer for A2 and B2 isomers were not observed when the L234 M mutation was combined with A1- and B1-producing KR types. For example, AcuM1 and MegM5, the two highest producing A1 KR exchanges, had product titers reduced by 67 and 65%, respectively, and the wild-type A1-type KR of Pik167 had titers reduced by 57%. These results indicate that the L234 M mutation changes the Pik167 KS to be more accepting of the A2- and B2-type intermediates, which have the α-methyl in the R-configuration, but at the cost of being more selective against the S-configuration.

The effects of the other mutations were less promising. Tryptophan 236 is associated with both A1- and B1-type KR domains while Pik127 has an alanine in the same position; therefore, we hypothesized that the W236A mutant could increase the titers of the A2- and B2-type epimerized products. However, in all cases, this was the worst performing point mutation and abolished almost all triketide lactone production (Figure S8). L234A was introduced to evaluate whether removing steric hindrances to the substrate tunnel could reduce stereochemistry-related gatekeeping by introducing a smaller alanine residue in place of the larger leucine residue. However, none of the tested KR exchanges made more of their product, except a small increase in the low producing HalM7 variant. The final mutation, L234T, is associated with A1 and B1-type stereochemistry. This mutation decreased activity of all exchanges, except BafM9 that was modestly increased. Since the second KS domain in Pik167 is already adapted for A1 substrates, it was unsurprising that this mutation offered no further improvement.

To investigate the utility of exchanging the entire functional AT-KR-ACP-KS evolutionary units, we selected one representative donor of each type, the same set of four donors that performed well in Lip1-TE and were used for Pik127 KR domain exchanges, and tested multiple exchange strategies: KR alone, KR-ACP, AT-KR-ACP, and each with the donor KS domain respectively (Figure 4c). The results from exchanging the KS domain along with the rest of the functional unit show that the approach can provide dramatic improvements in titers (up to 17-fold with LanM1) but also carries the significant risk of completely abolished reduction activity, as was the case with the A1-type AcuM1. The KR-only Acu-A1 exchange was highly successful, with titers of 406.2 mg/L, but in any case where the native KS was exchanged with the donor KS, all reduction activity was lost. Similarly, both FD8-A2 and Tyl-B1 donor multidomain exchanges showed reduced activity compared to their respective KR-only domain exchanges.

Conversely, the B2-type LanM1 exchange, which produced only 4.5 mg/L with the KR-only strategy, jumped to 77.6 mg/L when the full AT-KR-ACP-KS unit was exchanged, which is double the titer produced with the L234 M mutation. These data, along with the data from the point mutations, suggest that the native KS domain, which natively prefers the A1-type stereochemistry, strongly gatekeeps against the B2-type stereochemistry. These results suggest that while some KS domains are compatible with the Pik167 PKS, others may disrupt essential protein–protein interactions or cause structural instability. The compatibility between donor and acceptor systems appears to be a critical factor for the success of multidomain exchanges.

We also performed targeted proteomics on the Pik127 and Pik167 domain exchanges to investigate relationships between PKS expression and the measured titers (Figure S9). Protein levels were consistent across different domain exchange constructs, with most variants showing similar expression to the native pikromycin PKSs. This indicates that the observed differences in product titers primarily arise from changes in catalytic activity of the engineered PKS variants, rather than solely differences in protein abundance. Among all Pik127 and Pik167 strains, only the first polypeptide in Pik167 with the KR domain exchange data set was found to have a moderate correlation between protein abundance and production (Pearson *r*_*p*_ = 0.65, *p* = 0.004 and Spearman *r*_*s*_ = 0.27, *p* = 0.6), and even that relationship explained only ∼ 10% of the measured variance. Notably, AcuM1 achieved high titers with the KR-only strategy (406.2 mg/L) despite the protein levels being lower than the WT, while other variants, like MycM6, had higher protein levels with poor production. Additionally, the evolutionary unit exchanges that lost activity with KS domain exchanges maintained similar or even high protein expression levels, showing that the failure of these strains resulted from incompatible domain–domain interfaces or other factors, rather than poor expression alone. Conversely, the B2-type LanM1 AT-KR-ACP-KS domain exchange, which yielded a 17-fold improvement, did not achieve this performance through higher protein abundance, which likely indicates that this was through increased catalytic efficiency gained with compatibility of the domains. Combined with the Lip1-TE proteomics data, this points toward the conclusion that protein abundance alone cannot explain the difference in catalytic efficiency, and that some domain exchanges are intrinsically better performers.

## Discussion

In this study, we demonstrated that KR domain-regulated polyketide stereochemistry in PKSs can be systematically engineered to yield novel products. Since natural PKSs are involved in producing many pharmacologically relevant products, including several blockbuster drugs with sales over a billion dollars, engineering PKS stereocenters has the potential to produce polyketide stereoisomers with improved properties. Stereoisomers often exhibit dramatically different biological activities, and structural alterations could potentially enhance potency, reduce side effects, or increase metabolic stability.^44^ Another application is in the production of small molecules using PKSs with only a few modules. Since enzymes that act on small molecules typically display high stereoselectivity, precise control over PKS stereochemistry is essential for generating biologically active compounds compatible with downstream enzymatic processes.^45^ These therapeutic and biocatalytic applications require understanding how KR stereoselectivity can be predictably engineered, along with knowledge of how subsequent domains accommodate sterically altered intermediates without compromising the efficiency of the PKS.

For this study, we used domain exchange as a strategy to alter KR stereoselectivity, which offers distinctive advantages over point mutation approaches. While KR point mutations could potentially minimize structural disruptions that occur in whole KR domain exchanges, the complex interactions between the KR and other PKS domains remains insufficiently understood for reliable targeted mutagenesis. Whole-domain exchanges effectively alter both α- and β-substituents, but risk destabilizing the structure of the PKS through non-native protein–protein interactions. Previous work has demonstrated that altering the position of a PKS domain junction for a domain exchange by just a few amino acids can impact whether a chimeric PKS maintains full activity or loses all function, and careful optimization of these domain junctions can substantially alleviate any potential structural destabilization.^46^ Although our data confirm previous observations that KR domain exchanges can compromise PKS activity, we also showed that chimeric PKSs with donated KR domains sometimes outperformed the native KR.^26^ Replacing the native KR with more efficient donor KR domains improved the titers of Lip1-TE, with some variants producing up to 2.5 times the titers of 3-hydroxyacids compared the wild type. Some of the highest titers were from chimeric PKSs that generated predominantly ketone products at levels up to double the native system (133.1 compared to 63.1 mg/L). Though the inactivated variant, KR*, also increased overall PKS activity relative to the native, some donor KR domains had overall production much higher than achieved through simple inactivation, suggesting these domains contribute specific structural or catalytic properties that optimize the entire PKS system beyond simply changing the reduction state. These findings support the prospect of using domain exchanges as an approach for increasing polyketide production beyond native production levels.

Previous literature in KR domain exchanges has documented difficulties in switching the A-type to B-type KR domains.^25–30^ However, our exchange strategy produced high titers with both A- and B-type stereochemistry across all three PKS acceptors tested. In some cases, the unreduced ketone side product from nonfunctional KR domains was present in high titers, but this occurred across all four stereochemical configurations with KR domain exchanges from donors with low chemosimilarity rather than with any specific KR type. Our results suggest that α-substituent epimerization presents a greater challenge than β-hydroxyl stereochemistry. While Lip1-TE and Pik127 natively possess epimerization capabilities and readily accepted A2- and B2-type KRs, Pik167, which as an A1-type lacks native epimerization, often failed to correctly process substrates with an *R*-configured α-methyl substituent. This observation extends beyond Pik167, as similar challenges in introducing epimerizing KR domains have been reported in the A1-type DEBSM2 system, suggesting that the epimerization capability of the acceptor PKS is a critical factor in successful KR domain exchanges.^26,27,29^

Since downstream KS domains play an established role in gatekeeping against non-native intermediates, we investigated two different strategies to characterize and limit the gatekeeping against intermediates with altered stereochemistry using the two-module Pik167 PKS system.^41^ The first strategy relies on targeted KS point mutations, and the second on exchanging the entire Type I PKS evolutionary unit up to the donors’ corresponding KS domain. While previous literature has used point mutations to alter the general substrate specificity of a KS domain toward nonnative PKS intermediates, we identified our target residues through multiple sequence alignments, specifically seeking residues correlated with upstream KR domain types and their stereochemical preferences (Figure S10).^47–49^ One A2- and one B2-type KR donor domain functioned successfully in Pik167. The L234 M KS mutation, associated with upstream epimerizing KR domains, showed an improvement when paired with all four KR domains that successfully produced the epimerized product, and even significantly boosted the titers of two of them (by 1.7 and 8.1 times, respectively). However, there are inherent limitations, as the same mutation reduced the activity in nonepimerizing KR-types, showing the challenge of engineering a single active site to accommodate unnatural substrates while maintaining overall PKS function. These results confirm the potential of rational KS engineering using strategic point mutations, though additional studies with diverse PKS systems are needed to confirm the broader applicability of this approach and to identify other key residues involved in stereochemical gatekeeping.

In contrast, the approach of exchanging the entire evolutionary unit (AT-KR-ACP-KS) demonstrated the potential for more dramatic improvements, but only when the donor PKS works with the acceptor. The LanM1 variant had titers of 4.5 mg/L with the KR-only approach, but exchanging the entire AT-KR-ACP-KS functional unit increased the titers by 17-fold to 77.6 mg/L, which is more than double that of the KS point mutation alone. This could be because the coevolved domain units are more compatible and remove the gatekeeping bottlenecks associated with unnatural PKS contexts. However, this approach had higher risk than the KR-only approach, as some combinations, like with the Acu-A1 donor, abolished PKS activity entirely. Furthermore, exchanging the entire AT-KR-ACP-KS functional unit has the benefit of preserving the interactions of other domains with the KR, including the ACP and TE domains. Using point mutations to engineer these interfaces could further enhance the performance of chimeric PKSs containing nonnative KR domains, without risking breaking the entire PKS with a non-native KS domain exchange.^37,49,50^

Furthermore, targeted proteomics analysis across both the Lip1-TE system in *E. coli* K207–3 and the Pik systems in *S. albus* J 1074 revealed that measured improvements in titers likely primarily reflect intrinsic catalytic properties and domain compatibility rather than protein expression. Measured protein abundance showed that the correlation between titers and protein levels are limited, explaining only 24% of Lip1-TE and 10% of Pik167 of the differences in titers between the engineered variants relative to the WT PKS. Critically, the specific productivity of each variant, or titers normalized to protein abundance, had over 10-fold higher variation than protein expression alone, demonstrating that catalytic efficiency differences far exceed the contribution of expression levels to final titers. High performing donors achieved superior titers despite moderate protein levels, while variants with high protein expression were sometimes poor producers. These results demonstrate that catalytic efficiency and structural compatibility and not simply expression levels play a role in the success of a given KR domain exchange.

Based on the collective findings from this study, we can suggest practical recommendations for successful KR domain engineering in PKS systems. First, our investigation into additional factors that might influence KR domain exchanges revealed that maintaining the native DE does not affect titers in the one-module Lip1-TE system. Second, chemical similarity alone failed to reliably predict the success of a KR domain exchange. While donor KR domains with higher chemosimilarity to the Lip1-TE KR generally performed better, notable exceptions existed, underscoring the importance of other factors beyond simply substrate similarity. Third, exchanging only the KR domain, rather than multiple domains, gave higher success rates, possibly by minimizing disturbances in the structure of the protein. Our results also suggest that testing multiple donor KR domains with a new acceptor PKS is essential, as we found limited correlations between KR performance across PKS systems. Fourth, when using PKS systems that lack native epimerization, our data show that selecting A1- or B1-type KR donors typically results in more functional proteins that maintain the predicted stereochemical outcome, without requiring additional adaptations in the downstream domain. Finally, when engineering polyketides that require epimerization, two strategies can help overcome downstream gatekeeping, specifically whole functional unit exchange or targeted mutations in the downstream KS to improve acceptance of *R-*configured α-epimerized substituents. However, selecting a PKS platform that already supports epimerization may be the most reliable approach.

In this study, we have performed the systematic engineering of polyketide stereocenters through KR domain exchanges in three distinct PKS systems. By carefully optimizing domain boundaries, we successfully achieved all four possible stereochemical configurations in both single and two-module PKS systems, overcoming previous limitations in B-type stereochemistry exchanges. Furthermore, we demonstrated the critical role of the downstream KS domain in gatekeeping against non-native stereochemical configurations. Our targeted KS mutations, specifically the L234 M substitution, provided predictable improvements for epimerized intermediates, though with trade-offs between the four different stereochemical types. Our investigation of evolutionary functional unit exchanges revealed that exchanging entire AT-KR-ACP-KS blocks can seemingly overcome gatekeeping limitations within a host PKS, though this strategy does carry the risk of abolishing PKS activity. The ability to reliably engineer polyketide stereochemistry represents a significant advancement toward the rational design of novel bioactive compounds. By providing a systematic framework for KR domain exchanges and demonstrating the effectiveness of targeted KS modifications, this work establishes a more reliable foundation for engineering polyketides with precise stereochemical control.

## Methods

### Chemicals and Reagents

All chemicals were purchased from Sigma-Aldrich (USA) unless otherwise noted.

Phanta Max DNA polymerase was purchased from Vazyme (China). All primers were synthesized by Integrated DNA Technologies (USA). NEBuilder HiFi DNA Assembly Master Mix and One*Taq* DNA polymerase for genomic PCRs were purchased from New England Biolabs (USA). Plasmids were purified using the QIAprep Spin Miniprep Kit and DNA fragments were purified using the QIAquick Gel Extraction Kit from Qiagen (USA).

(2*S*,3*S*)-3-hydroxy-2,4-dimethylpentanoic acid (the A2-type Lip1-TE product) was purchased from Organic Consultants Incorporated. (2*R*,3*S*)- and (2*R*,3*R*)-3-hydroxy-2,4-dimethylpentanoic acid (the A1- and B1-type products respectively) were synthesized by Enamine (Ukraine). Ketone standards, including 3-pentanone, 2-hexanone, 3-hexanone, 2-methylpenta-3-one, and 4-methylpentane-3,5-dione were purchased from Thermo Fisher Scientific (USA).

### Media and Cell Cultivation

Overnight cultures of *E. coli* were inoculated into LB (Lennox) medium and shaken at 225 rpm at 37 °C. The medium contained either 50 mg/L kanamycin, 50 mg/L streptomycin, 25 mg/L apramycin, or 15 mg/L chloramphenicol, with the appropriate antibiotic(s) for all strains in Table 1, with the exception of kanamycin and streptomycin, which were used in half the concentration (25 mg/L) for both cultivating transformed *E. coli* ET12567 and for *E. coli* K207–3 during production runs. All *E. coli* cultures were grown in 3 mL in 24-well plates from VWR.

**Table 1:** Plasmids Used in This Study, along with the Part IDs for Each Strain Generated in This Study

plasmid	description	reference	JBEI part ID
ptm2	first polypeptide of Pik127	Miyazawa^40^ Ptm2, ptm3, ptm4, and ptm5 were kindly provided by the Keatinge-Clay lab	JBx_204244
ptm3	second polypeptide of Pik127	-	JBx_204245
ptm4	first polypeptide of Pik167	-	JBx_204246
ptm5	second polypeptide of Pik167	-	JBx_204247
p21	mCherry under gapdh promoter	Phelan et al.^52^	JBx_107121
p33	Lip1-TE	Yuzawa et al.^53^	JBx_134199
pSY188	KR* knockout for ketone production	Yuzawa et al.^31^	JBx_083451
p33_AcuM1_AT-KR-ACP	aculeximycin AT-KR-ACP swap from module 1	this work	JBx_193037
p33_AcuM1_KR-ACP	aculeximycin KR-ACP swap from module 1	this work	JBx_193038
p33_AcuM1_AT-KR	aculeximycin AT-KR swap from module 1	this work	JBx_193039
p33_AcuM1_KR	aculeximycin KR swap from module 1	this work	JBx_193040
p33_BafM1_AT-KR-ACP	bafilomycin AT-KR-ACP swap from module 1	this work	JBx_193041
p33_BafM1_KR-ACP	bafilomycin KR-ACP swap from module 1	this work	JBx_193042
p33_BafM1_AT-KR	bafilomycin AT-KR swap from module 1	this work	JBx_193043
p33_BafM1_KR	bafilomycin KR swap from module 1	this work	JBx_193044
p33_DEBSM1_AT-KR-ACP	erythromycin AT-KR-ACP swap from module 1	this work	JBx_193045
p33_DEBSM1_KR-ACP	erythromycin KR-ACP swap from module 1	this work	JBx_193046
p33_DEBSM1_AT-KR	erythromycin AT-KR swap from module 1	this work	JBx_193047
p33_DEBSM1_KR	erythromycin KR swap from module 1	this work	JBx_193048
p33_TylKR1	tylactone KR1	this work	JBx_193049
p33_AveKR1	avermectin KR1	this work	JBx_193050
p33_ChaKR1	chalcomycin KR1	this work	JBx_193052
p33_MeiKR1	meilingmycin KR1	this work	JBx_193053
p33_AldKR2	aldgamycin KR2	this work	JBx_193054
p33_NemKR2	nemadectin KR2	this work	JBx_193055
p33_PM1KR4	PM100117 KR4	this work	JBx_193056
p33_HerKR6	herboxidiene KR6	this work	JBx_193057
p33_AcuKR8	aculeximycin KR8	this work	JBx_193058
p33_NemKR12	nemadectin KR12	this work	JBx_193059
p33_AcuKR15	aculeximycin KR15	this work	JBx_193060
p33_AmpKR1	amphotericin KR1	this work	JBx_193061
p33_PM1KR1	PM100117 KR1	this work	JBx_193062
p33_FD8KR2	FD-891 KR2	this work	JBx_193063
p33_StrKR4	streptolydigin KR4	this work	JBx_193064
p33_LasKR5	lasalocid KR5	this work	JBx_193065
p33_BafKR9	bafilomycin KR9	this work	JBx_193066
p33_SalKR14	salinomycin KR14	this work	JBx_193067
p33_MegKR1	megalomicin KR1	this work	JBx_193068
p33_LanKR1	lankamycin KR1	this work	JBx_193069
p33_FK5KR2	FK520 KR2	this work	JBx_193070
p33_AntKR2	antalid KR2	this work	JBx_193071
p33_AcuKR5	aculeximycin KR5	this work	JBx_193072
p33_HalKR7	halstoctacosanolide KR7	this work	JBx_193073
p33_MerKR10	meridamycin KR10	this work	JBx_193074
p33_AveKR1_DE2	AveKR1 with the native Lip1-TE DE instead	this work	JBx_193075
p33_ChaKR1_DE2	ChaKR1 with the native Lip1-TE DE instead	this work	JBx_193076
p33_BafKR1_DE2	BafKR1 with the native Lip1-TE DE instead	this work	JBx_193077
p33_PM1KR4_DE2	PM1KR1 with the native Lip1-TE DE instead	this work	JBx_193078
ptm5-L234A	ptm5 derivative with KS mutation L234A	this work	JBx_255633
ptm5-L234M	ptm5 derivative with KS mutation L234M	this work	JBx_255634
ptm5-L234T	ptm5 derivative with KS mutation L234T	this work	JBx_255635
ptm5-W236A	ptm5 derivative with KS mutation W236A	this work	JBx_255636
ptm2-AcuM1	aculeximycin KR1	this work	JBx_265864
ptm2-FD8M2	FD-891 KR2	this work	JBx_265865
ptm2-TylM1	tylactone KR1	this work	JBx_265866
ptm2-LanM1	lankamycin KR1	this work	JBx_265867
ptm4-AcuM1	aculeximycin KR1	this work	JBx_265823
ptm4-AngM1	angelomycin KR1	this work	JBx_265824
ptm4-Meg5	megalomicin KR5	this work	JBx_265825
ptm4-Myc6	mycinamicin KR6	this work	JBx_265826
ptm4-FD8M2	FD-891 KR2	this work	JBx_265827
ptm4-StrM4	Streptolydigin KR4	this work	JBx_265828
ptm4-LasM5	lasalocid KR5	this work	JBx_265829
ptm4-BafM9	bafilomycin KR9	this work	JBx_265830
ptm4-TylM1	tylactone KR1	this work	JBx_265831
ptm4-AldM2	aldgamycin KR2	this work	JBx_265832
ptm4-PM1M4	PM100117 KR4	this work	JBx_265833
ptm4-NemM12	nemadectin KR12	this work	JBx_265834
ptm4-LanM1	lankamycin KR1	this work	JBx_265835
ptm4-AntM2	antalid KR2	this work	JBx_265836
ptm4-HalM7	halstoctacosanolide KR7	this work	JBx_265837
ptm4-MerM10	meridamycin KR10	this work	JBx_265838
ptm5-AcuKS	ptm5 with KS from AcuM1	this work	JBx_275424
ptm5-FD8KS	ptm5 with KS from FD8M2	this work	JBx_275429
ptm5-TylKS	ptm5 with KS from TylM1	this work	JBx_275437
ptm5-LanKS	ptm5 with KS from LanM1	this work	JBx_275434
ptm4-AcuKR-ACP	AcuM1 didomain exchange	this work	JBx_275426
ptm4-AcuAT-KR-ACP	AcuM1 tridomain exchange	this work	JBx_275432
ptm4-FD8KR-ACP	FD8M2 didomain exchange	this work	JBx_275431
ptm4-FD8AT-KR-ACP	FD8M2 tridomain exchange	this work	JBx_275433
ptm4-TylKR-ACP	TylM1 didomain exchange	this work	JBx_275438
ptm4-TylAT-KR-ACP	TylM1 tridomain exchange	this work	JBx_275439
ptm4-LanKR-ACP	LanM1 didomain exchange	this work	JBx_275435
ptm4-LanAT-KR-ACP	LanM1 tridomain exchange	this work	JBx_275436

*S. albus* J1074 strains were inoculated from Mannitol Soy agar plates with 10 mM magnesium chloride from Teknova into Trypic Soy Broth Dehydrated Culture Medium (TSB) containing 25 mg/L of nalidixic acid and 25 mg/L apramycin only for conjugated strains and incubated at 30 °C for 3 days. To analyze 3-hydroxyacid or unreduced product production by engineered *S. albus* J1074, it was grown in 042 medium (10 g/L glucose, 10 g/L corn starch, 10 g/L glycerol, 2.5 g/L solid corn steep, 5 g/L peptone, 2 g/L yeast extract, 1 g/L NaCl, and 3 g/L CaCO_3_ adjusted to pH 7.2).^51^ Production was allowed to continue for 8 days at 30 °C in 20 mL medium in 250 mL flasks, as described previously.^31^

To analyze triketide lactone or unreduced or incomplete product production by engineered *E. coli* K207-3, it was inoculated at 5% with washed overnight cultures, into either LB medium, the production medium described by Miyazawa et al. (5 g/L yeast extract, 10 g/L casein, 15 g/L glycerol, 10 g/L NaCl, and 100 mM potassium phosphate set to pH 7.6),^40^ or EZ Rich defined medium from Teknova, with the appropriate halved concentrations of antibiotics (Figure S11). Once grown to an OD600 of ∼0.6, the temperature was cooled to 18 °C, and production was induced with 0.1 mM IPTG and 20 mM sodium propionate; the cultures were grown for 5 days.

### Strains and Plasmids

Plasmids and strains used in this study are found in Table 1 and Table 2 and are publicly available through the JBEI registry (https://public-registry.jbei.org/folders/900). All KR domains from donor PKSs were synthesized by Twist Biosciences after undergoing codon optimization for *E. coli* expression with IDT’s codon optimization tool and having the rare *Streptomyces sp.* TTA codon removed.

**Table 2:** Strains Used in This Study Are Listed, Along with the Reference Numbers for Original Strains

strains	description	reference	selection
*E. coli* ET12567	*Streptomyces* sp. conjugation strain	ATCC BAA-52535 ^54^	CmR, KanR
*E. coli* K207–3	Engineered host for pikromycin PKSs	Murli et al.^55^	NxR
*Streptomyces albus* J1074		Zaburannyi et al.^56^	NalR

### Conjugation of Vectors into *Streptomyces albus* J1074

Vectors were conjugally transferred into *Streptomyces albus* J1074 based on the previously described method.^31^ In short, conjugation plasmids were transformed into *E. coli* ET12567/pUZ8002, and the resulting transformants were seeded into 10 mL LB at 37 °C with the appropriate antibiotic. Once the cultures reached an OD_600_ of 0.4–0.6, the *E. coli* were pelleted, washed with 500 μL LB two times, and then finally was resuspended in 400 μL LB. Meanwhile, the spores from a fresh, fully grown plate of *S. albus* on MS agar was gathered with 5 μL of sterile water, and 500 μL of the spores were mixed with the 500 μL *E. coli*. The mixture was pelleted and plated on MS agar at 30 °C, and the next day an overlay of nalidixic acid and apramycin in a 1 mL mixture was added to the plate, which was incubated longer until colonies appeared. Conjugal transfer of all vectors was confirmed via genomic PCR.

### Synthesis of Authentic Enantiomerically Pure Triketide Lactone Standards

The four enantiomerically pure triketide lactone standards were prepared by organic chemistry. The detailed methods and NMR spectra can be found in the Supporting Information.

### Harvesting Lip1-TE Products from *S. albus* J1074

Lip1-TE produces the 3-hydroxyacid (2*S*,3*S*)-3-hydroxy-2,4-dimethylpentanoic acid with a native A2-type KR domain, while a lack of reduction produces 2-methylpentan-3-one ketone. LC-MS samples for quantifying 3-hydroxy-2,4-dimethylpentanoic acid were harvested by mixing 500 μL of the supernatant with 500 μL methanol, then spun through a 350 μL 3K MWCO AcroPrep Advance 96-well filter plate from Pall Lab (USA) before being transferred to GC vials for further analysis. 200 μL of sample supernatant was set aside for ketone quantification via GC-MS.

For stereochemistry measurements on the GC-MS, the 3-hydroxyacid was derivatized to a methyl-ester by drying 200 μL of supernatant in a SpeedVac (SPD111 V) from ThermoFisher Scientific at 60 °C for 2 h, and then resuspending in 200 μL of analytical-grade methanol with 10 μL of 72% H_2_SO_4_. The screwcap tubes were incubated at 90 °C in a tabletop shaker at 1000 rpm for 90 min for derivatization. After addition of 100 μL water and 200 μL ethyl acetate, the organic layer was removed for GC-MS analysis.

### LC-MS Quantification of 3-Hydroxy-2,4-dimethylpentanoic Acid

3-Hydroxy-2,4-dimethylpentanoic acid was quantified via LC-MS against authentic standards using methods similar to those previously described.^57^ In short, LC-MS analysis was conducted on a Kinetex XB-C18 column (100 mm length, 3.0 mm internal diameter, and 2.6-μm particle size; Phenomenex, Torrance, CA USA) using a 1260 Infinity HPLC system (Agilent Technologies, Santa Clara, CA, USA). A sample injection volume of 3 μL was used throughout. The sample tray and column compartment were set to 6 and 25 °C, respectively. The mobile phase was composed of 0.1% formic acid in water (solvent A) and 0.1% formic acid in methanol (solvent B), unless stated otherwise. 3-hydroxyacids were separated via gradient elution under the following conditions: linearly increased from 20% B to 72.1% B in 6.5 min, linearly increased to 95% B in 1.3 min, then held at 95% B for 2 min, linearly decreased from 95% B to 20% B in 0.2 min, and held at 5% B for 2.2 min. The flow rate was held at 0.42 mL/min for 8.8 min, linearly increased from 0.42 mL/min to 0.65 mL/min in 0.2 min, and held at 0.65 mL/min for 2.2 min. The total LC run time was 11.2 min. The HPLC system was coupled to an Agilent Technologies 6520 quadrupole time-of-flight mass spectrometer (for LC-QTOF-MS) via a 1:4 postcolumn split. ESI was conducted in the negative ion mode and a capillary voltage of 3,500 V was utilized. The data acquisition range was from 50 to 500 *m*/*z*, and the acquisition rate was 0.86 spectra/s. Data acquisition (Workstation B.08.00) and processing (Qualitative Analysis B.06.00 and Profinder B.08.00) were conducted via the Agilent MassHunter software package.

### Measuring the Stereochemistry of 3-Hydroxy-2,4-dimethylpentanoic Acid

Samples for stereochemical analysis were performed similarly to previously described.^28^ In brief, 1 μL of each sample was injected into an Agilent 6890 gas chromatograph equipped with a 30 m × 0.25 mm, 0.25 μm CycloSil-B chiral capillary column from Agilent, followed by an Agilent 5973 mass detector. Mass fragments and retention times were compared to the enantiomerically pure analytical standards.

### Proteomics Analysis

Protein was extracted from *E. coli* cell pellets and tryptic peptides were prepared by following established proteomic sample preparation protocol.^58^ Briefly, cell pellets were resuspended in Qiagen P2 Lysis Buffer (Qiagen, Germany) to promote cell lysis. Proteins were precipitated with addition of 1 mM NaCl and 4 × vol acetone, followed by two additional washes with 80% acetone in water. The recovered protein pellet was homogenized by pipetting mixing with 100 mM ammonium bicarbonate in 20% methanol. Protein concentration was determined by the DC protein assay (BioRad, USA). Protein reduction was accomplished using 5 mM tris 2-(carboxyethyl)­phosphine (TCEP) for 30 min at room temperature, and alkylation was performed with 10 mM iodoacetamide (IAM; final concentration) for 30 min at room temperature in the dark. Overnight digestion with trypsin was accomplished with a 1:50 trypsin:total protein ratio. The resulting peptide samples were analyzed on an Agilent 1290 UHPLC system coupled to a Thermo Scientific Orbitrap Exploris 480 mass spectrometer for discovery proteomics.^59^ Briefly, peptide samples were loaded onto an Ascentis ES-C18 Column (Sigma–Aldrich, USA) and were eluted from the column by using a 10 min gradient from 98% solvent A (0.1% FA in H2O) and 2% solvent B (0.1% FA in ACN) to 65% solvent A and 35% solvent B. Eluting peptides were introduced to the mass spectrometer operating in positive-ion mode and were measured in data-independent acquisition (DIA) mode with a duty cycle of 3 survey scans from *m*/*z* 380 to *m*/*z* 985 and 45 Tandem mass spectrometry (MS2) scans with precursor isolation width of 13.5 *m*/*z* to cover the mass range. DIA raw data files were analyzed by an integrated software suite DIA-NN.^60^ The database used in the DIA-NN search (library-free mode) is *E. coli* latest Uniprot proteome FASTA sequences plus the protein sequences of the heterologous proteins and common proteomic contaminants. DIA-NN determines mass tolerances automatically based on first pass analysis of the samples with automated determination of optimal mass accuracies. The retention time extraction window was determined individually for all MS runs analyzed via the automated optimization procedure implemented in DIA-NN. Protein inference was enabled, and the quantification strategy was set to Robust LC = High Accuracy. Output main DIA-NN reports were filtered with a global false discovery rate set at 0.01 (FDR ≤ 0.01) on both the precursor level and protein group level. The Top3 method, which is the average MS signal response of the three most intense tryptic peptides of each identified protein, was used to plot the quantity of the targeted proteins in the samples.^61^ A jupyter notebook written in Python executed label-free quantification (LFQ) data analysis on the DIA-NN peptide quantification report, and the details of the analysis were described in the established protocol.^62^

The generated mass spectrometry proteomics data have been deposited to the ProteomeXchange Consortium via the PRIDE partner repository with the data set identifier PXD066826.^63^ DIA-NN is freely available for download from https://github.com/vdemichev/DiaNN.

### Expression of Pikromycin PKS in *E. coli* K207-3

*E. coli* K207-3 with the appropriate two-plasmid system were grown in 3 mL LB at 30 °C overnight, then washed twice with 3 mL sterile water, before being seeded at 1% into the associated media. At OD_600_, expression was induced with 0.1 mM IPTG and 20 mM sodium propionate and grown for 5 days at 19 °C.^40^

Samples for LC-MS quantification and characterization were harvested by mixing 500 μL supernatant with 500 μL acetonitrile containing 100 μM triacetic acid lactone (TAL), and incubating at room temperature for 1 h. The samples were spun through the 3K AcroPrep filter plate, and the filtrate was transferred to GC vials for LC-MS analysis. 200 μL supernatant from each sample was set aside for GC-MS quantification of ketone byproducts.

### Stereochemistry Measurement of Triketide Lactones and Quantification of Related Products

LC-MS characterization of triketide lactone products and the related 3-hydroxyacid were run on a 1260 Infinity II LC/MSD XT (Agilent) LC-MS, through the Lux 3 μM 150 mm × 4.6 mm Cellulose-1 LC column (Phenomenex) at room temperature. Ten uL was injected for all runs. LC-MS grade water and methanol with 0.1% formic acid (buffers A and B respectively) were run with the following gradient at 1.4 mL/min: Linearly increased buffer B from 20 to 45% over 15.50 min, dropped back from 45 to 20% over 0.30 min, then held at 20% for 2.20 min, for a total of 18 min. Identification of analytes was confirmed with mass spectra gathered with electrospray ionization (ESI) in positive and negative scanning mode over the range of mass-to-charge ratio *m*/*z* 100 to *m*/*z* 200. The mass and retention times of all samples were compared to the authentic standards and quantified with a standard curve containing the appropriate internal standard.

### Quantification of Ketone Incomplete Reduction Byproducts

Ketones were measured on the GC-MS as previously described.^31^ In short, 200 μL of supernatant was incubated with an equal volume of methanol in 2 mL screwcap tubes at 50 °C overnight for decarboxylation. 2-Methylpenta-3-one was extracted with hexane containing an internal standard of 50 mg/L 3-hexanone, and 3-pentanone was extracted with pentane containing an internal standard of 5 mg/L (50 uM) 2-hexanone. Extracted ketones in the organic phase were transferred to GC vials for quantification. Samples were measured on a Agilent Intuvo 9000 GC-MS system equipped with an Agilent DB-WAX UI column with dimensions 15 m × 0.25 mm, 0.25 μm in length. One μL of the ketone samples were injected at 1 mL/min with an inlet temperature of 250 °C, and the oven was held at 50 °C for 5 min followed by a ramp of 100 °C/min to 250 °C. Standard curves for quantification of analytes and confirmation of retention times were produced using analytical-grade standards, along with the associated internal standard.

### Chemical and Sequence Similarity Analysis

Chemical structure comparison was performed by converting each structure into SMILES representations then calculating the Tanimoto similarity in Python 3.8.16 using RDKit v2024.03.5. Protein sequence comparison for the KR domains was performed using the pairwise aligner from Biopython v1.78. All code used to perform this analysis is publicly available on GitHub (https://github.com/Keasling-Lab/KR_swap).

### Structural Comparison of Lip1-TE and Pik167 KRs to Donor KRs

Protein structures for KR and KS domains were predicted using AlphaFold2 via ColabFold v1.5.5. Each PDB file was superimposed onto the structural prediction of the reference domain from either Lip1 or Pik167 using the PDB module from Biopython v1.78 to calculate the root-mean-square deviation between the superimposed coordinates.

## Supplementary Material



## Notes

PKS domain boundaries; standard curves used for quantification; GC-MS chiral column measurements for 3-hydroxy-2,4-dimethylpentanoic acid enantiomers; KR domains, SMILEs, and types; retention times of GC-MS column measurements for Lip1-TE PKS variants; chemical and sequence similarity rankings for KR donors relative to acceptor KR domains; proteomics measurements for Lip1-TE (inserted); KR inactivation mutation for ketone production; LC-MS separation of triketide lactone enantiomers; retention times of LC-MS column measurements for Pik167 PKS variants; each KR domain exchange with each of the four KS point mutations; proteomics measurements for Pik127 and Pik167; KS point mutations in Pik167; PKS production media for the Pik127 and Pik167 systems; and NMR and other supporting experiments and measurements (PDF)

## Abbreviations

PKS: polyketide synthase
KR: ketoreductase
AT: acyltransferase
ACP: acyl carrier protein
KS: ketosynthase
TE: thioesterase
DH: dehydratase
ER: enoylreductase
DE: dimerization element
