# Supplementary material for: Engineering Polyketide Stereocenters with Ketoreductase Domain Exchanges
Source: J Am Chem Soc. 2025 Nov 4;147(46):42237–52. doi: 10.1021/jacs.5c06736 (PMC12636015; doi:10.1021/jacs.5c06736)
Supplement: Supplementary file 1 [file ja5c06736_si_001.pdf]

Engineering polyketide stereocenters with ketoreductase domain exchanges

Leah S. Keiser[1,2,3], Panarai Primrose Gatenil[2,3,4], Yolanda Zhu[1,2,3], Kai Deng[1,5], Lucas Waldburger[1,3,4,6], Jennifer W. Gin[1], Yan Chen[1], Edward E. K. Baidoo[1,3], Christopher J. Petzold[1], Nathan Lanclos[1,3,4], Trent R. Northen[1,6], Elias Englund[1,7,*], Jay D. Keasling[1,2,3,4,8,9,*]

[1]Joint BioEnergy Institute, 5885 Hollis Street, Emeryville, CA 94608, USA
[2]Department of Chemical and Biomolecular Engineering, University of California, Berkeley, CA 94720, USA
[3]Biological Systems & Engineering Division, Lawrence Berkeley National Laboratory, Berkeley, CA 94720, USA
[4]Department of Bioengineering, University of California, Berkeley, CA 94720, USA
[5]Department of Biomaterials and Biomanufacturing, Sandia National Laboratories, Livermore, CA 94550, USA
[6]Environmental Genomics and Systems Biology Division, Lawrence Berkeley National Laboratory, Berkeley, CA 94720, USA
[7]School of Engineering Sciences in Chemistry, Biotechnology and Health, Science for Life Laboratory, KTH – Royal Institute of Technology, 106 91 Stockholm, Sweden
[8]California Institute for Quantitative Biosciences (QB3), University of California, Berkeley, CA 94720, USA
[9]The Novo Nordisk Foundation Center for Biosustainability, Technical University of Denmark, Lyngby 2800, Denmark

*Co-corresponding authors: Elias Englund, elias.englund@scilifelab.se, and Jay D. Keasling, jdkeasling@lbl.gov

AUTHOR ADDRESS: 5885 Hollis Street, Emeryville, CA 94608, USA

Supplementary information

Figure S1: PKS domain boundaries

Figure S2: Standard curves used for quantification

Figure S3: GC-MS chiral column measurements for 3-hydroxy-2,4-dimethylpentanoic acid enantiomers

Table S1: KR domains, SMILES, and types

Table S2: Retention times of GC-MS column measurements for Lip1-TE PKS variants

Figure S4: Chemical and sequence similarity rankings for KR donors relative to acceptor KR domains

Figure S5: Proteomics measurements for Lip1-TE (inserted)

Figure S6: KR inactivation mutation for ketone production

Figure S7: LC-MS separation of triketide lactone enantiomers

Table S3: Retention times of LC-MS column measurements for Pik167 PKS variants

Figure S8: Each KR domain exchange with each of the four KS point mutations

Figure S9: Proteomics measurements for Pik127 and Pik167

Figure S10: KS point mutations in Pik167

Figure S11: PKS production media for the Pik127 and Pik167 systems

Triketide lactone synthesis and NMR spectra: Synthesis of triketide lactones

PKS boundaries

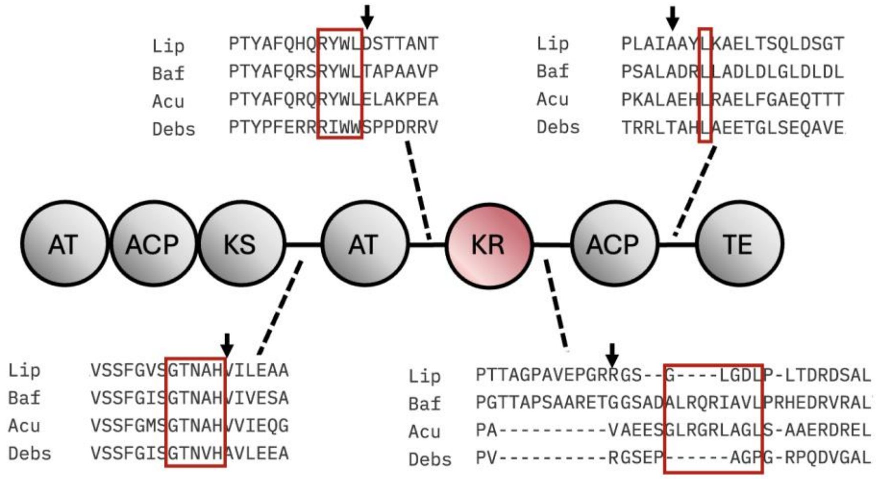

Figure S1: The KS-AT junction was placed after the GTNAH motif to maintain the KS and AT linker (KAL), and the AT-KR junction was placed after the post AT linker (PAL) marked by the RYWL sequence as previously described by Yuzawa et al.[1] The KR-ACP boundary was defined by the GLGDL sequence, as described in previous Lip1-TE KR domain exchanges.[2] The end of the ACP domain was delineated by the conserved L residue corresponding to the final α-helix of the ACP.[3,4] KR domains with a donor DE also used the RYWL motif as the starting boundary, while exchanges retaining the native Lip1-TE used the end of the DE sequence as the boundary in both the acceptor and donor PKSs.

Standard curves for quantification

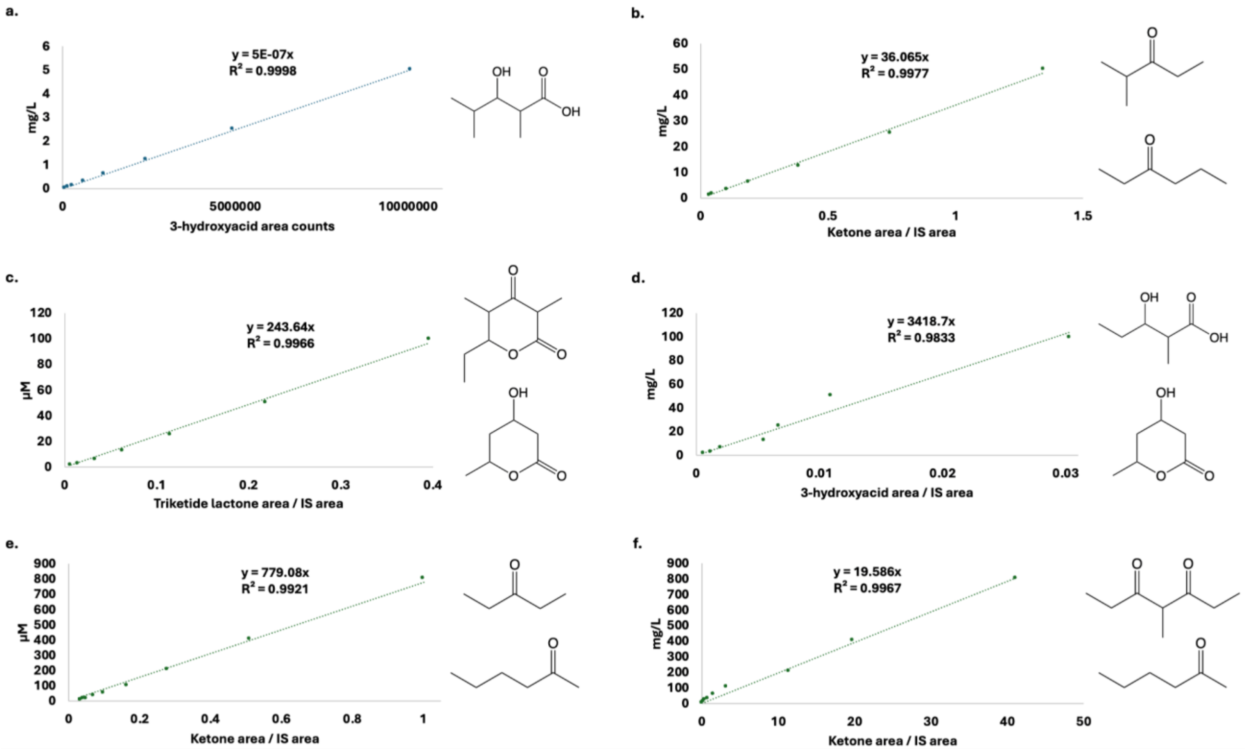

Figure S2: The standard curves used for quantification of a) 3-hydroxy-2,4-dimethylpentanoic acid produced by Lip1-TE b) 2-methylpentan-3-one produced by Lip1-TE against a 3-hexanone internal standard (IS) c) 6-ethyl-3,5-dimethyldihydro-2*H*-pyran-2,4(3*H*)-dione (triketide lactone) produced by Pik127 and Pik167 against a triacetic acid lactone IS d) 3-hydroxy-2-methylpentanoic acid produced by Pik127 and Pik167 against a triacetic acid lactone IS and e) The one-extension unreduced 3-pentanone produced by Pik127 and Pik167 against a 2-hexanone IS. f) The 4-methylheptane-3,5-dione derived from the two-extension unreduced ketone product was measured against a 2-hexanone IS. This product was not detected in any samples.

KR type, DE sequences, and SMILES

Table S1: Listed are the KR domain donors used in this study for Lip1-TE, Pik127, and Pik167 KR domain exchanges, along with their type, abbreviated identifier, whether or not they contain a native dimerization element (DE), and SMILES string for their native substrates. The full sequences are available at JBEI REGISTRY (**Tables 1 and 2**).

Abbreviated name	Swapped to Lip1-TE	Swapped to Pik167	KR donor	Module	DE	KR type	SMILES
Lip1	-		Lip1-TE	1	yes	A2	CC(C)[C@H](O)[C@H](C)C(=O)[S]
Pik5		-	Pik167	5	no	A1	CC[C@@H](O)[C@H](C)C=[C]C(=O)[C@H](C)[CH][C@H](C)[C@H](O)[C@@H](C)C(=O)[S]
Pik1	-	-	Pik127	1	no	B2	CC[C@@H](O)[C@H](C)C(=O)[S]
Amp1	yes		Amphotericin	1	yes	A2	C[C@H](O)[C@H](C)C(=O)[S]
PM11	yes		PM100117	1	yes	A2	CC[C@@H]([C@@H](C([S])=O)C)O
FD82	yes	yes	FD-891	2	yes	A2	C[C@H](C(=O)[S])[C@@H](O)[C@H](C)[C@H](C)O
Str4	yes	yes	Streptolydigin	4	yes	A2	CC(=O)[C@@H](C)[C@@H](O)CC=C(C)[C@H](O)[C@H](C)C(=O)[S]
Las5	yes	yes	Lasalocid	5	yes	A2	CC=C([CH][CH]C=C([CH][C@H](C)[C@H](O)[C@H](CC)C(=O)[S])CC)CC
Baf9	yes	yes	Bafilomycin	9	yes	A2	CO[C@@H](C=[C]C=C(C)[CH][C@H](C)[C@H](O)[C@H](C)C(=O)[S])[C@H](O)[C@@H](C)[C@@H](O)[C@H](C)C(=O)C[C@@H](O)[C@H](C)[C@H](O)C(C)C
Sal14	yes		Salinomycin	14	yes	A2	CC=C([CH][CH]C=C(C)[CH][CH]C(=O)C[C@H](O)CC(=O)[C@@H](C)[CH][C@H](C)C(O)C(CC)C(=O)[C@@H](C)[C@@H](O)[C@H](C)[C@H](O)[C@H](C)[CH][CH][C@H](O)[C@H](CC)C(=O)[S])CC
Baf1	yes		Bafilomycin	1	yes	B1	CC(C)[C@@H](O)[C@@H](C)C(=O)[S]
Tyl1	yes	yes	Tylactone	1	no	B1	CC[C@@H](O)[C@@H](C)C(=O)[S]
Ave1	yes		Avermectin	1	yes	B1	CC(C)[C@H]([C@H](C([S])=O)C)O
Cha1	yes		Chalcomycin	1	no	B1	C[C@@H](O)[C@@H](C)C(=O)[S]
Mei1	yes		Meilingmycin	1	yes	B1	C[C@@H](O)[C@@H](C)C(=O)[S]
Ald2	yes	yes	Aldgamycin	2	no	B1	CC(=O)[C@H](C)C(O)C(C)C(=O)[S]

Nem2	yes		Nemadectin	2	yes	B1	CCC(C)[C@@H](O)[C@@H](C)[C@@H](O)[C@@H](C)C(=O)[S]
Pm14	yes	yes	PM100117	4	yes	B1	O[C@@H]([C@H](C([S])=O)C)C(C)C(O)C(C)C([C@H]([C@@H](O)CC)C)O
Her6	yes		Herboxidiene	6	yes	B1	CC[C@H](O)[C@@H](C)C=C(C)[CH][C@H](C)C=[C]C=C(C)[C@@H](O)[C@@H](C)C(=O)[S]
Acu8	yes		Aculeximycin	8	yes	B1	CC[C@H](O)[C@@H](C)[CH][CH][C@@H](O)C[CH][CH][C@@H](O)[C@H](C)C=[C]C=[C][C@@H](O)[C@@H](C)C(=O)[S]
Nem12	yes	yes	Nemadectin	12	yes	B1	CCC(C)[C@@H](O)[C@@H](C)[C@@H](O)[C@@H](C)[CH][CH]C(=O)C[C@@H](O)C[C@H](O)CC=C(C)[CH][C@H](C)C=[C]C=C(C)[C@@H](O)C[C@@H](O)[C@@H](C)C(=O)[S]
Acu15	yes		Aculeximycin	15	yes	B1	CC[C@H](O)[C@@H](C)[CH][CH][C@@H](O)C[CH][CH][C@@H](O)[C@H](C)C=[C]C=[C][C@@H](O)[C@@H](C)[C@H](O)CC(O)C[C@H](O)[C@@H](C)[C@@H](O)C[C@H](O)CC(=O)C[C@@H](O)[C@@H](C)C(=O)[S]
Debs	yes		Erythromycin	1	no	B2	CC[C@@H](O)[C@H](C)C(=O)[S]
Meg1	yes		Megalomicin	1	no	B2	CC[C@@H](O)[C@H](C)C(=O)[S]
Lan1	yes	yes	Lankamycin	1	no	B2	CCC(C)[C@@H](O)[C@H](C)C(=O)[S]
FK52	yes		FK520	2	yes	B2	CC(=CC1CC[C@@H](O)[C@H](O)C1)[C@@H](O)[C@H](C)C(=O)[S]
Ant2	yes	yes	Antalid	2	yes	B2	O[C@@H]([C@@H](C([S])=O)C)C[C@@H](O)CC
Acu5	yes		Aculeximycin	5	yes	B2	CC[C@H](O)[C@@H](C)[CH][CH][C@@H](O)C[CH][CH][C@@H](O)[C@H](C)C(=O)[S]
Hal7	yes	yes	Halstoctacosanolide	7	yes	B2	CC[C@H](O)[C@@H](C)[C@H](O)C[C@@H](O)CC=C(C)[CH][C@H](C)[C@H](O)C[C@@H](O)[C@H](C)C(=O)[S]
Mer10	yes	yes	Meridamycin	10	yes	B2	CCC(=CC[C@H](O)C(C)=C[C@H](C)[C@H](C)O)C(=O)[C@H](C)[CH][C@H](C)C=C(C)[C@H](O)C[C@@H](O)C[C@@H](O)[C@H](C)C(=O)[S]
Acu1	yes	yes	Aculeximycin	1	yes	A1	CC[C@H](O)[C@@H](C)C(=O)[S]
Ang1		yes	Angolamycin	1	no	A1	CC[C@H](O)[C@@H](C)C(=O)[S]
Meg5		yes	Megalomicin	5	no	A1	CC[C@@H](O)[C@H](C)[C@H](O)[C@@H](C)C(=O)[C@H](C)[CH][C@H](C)[C@H](O)[C@@H](C)C(=O)[S]
Myc6		yes	Mycinamicin	6	no	A1	CC[C@H](O)[C@@H](C)C=[C]C=[C]C(=O)[C@H](C)[CH][C@H](C)[C@H](O)[C@@H](C)C(=O)[S]

Chiral column measurements for Lip1-TE products on GC-MS

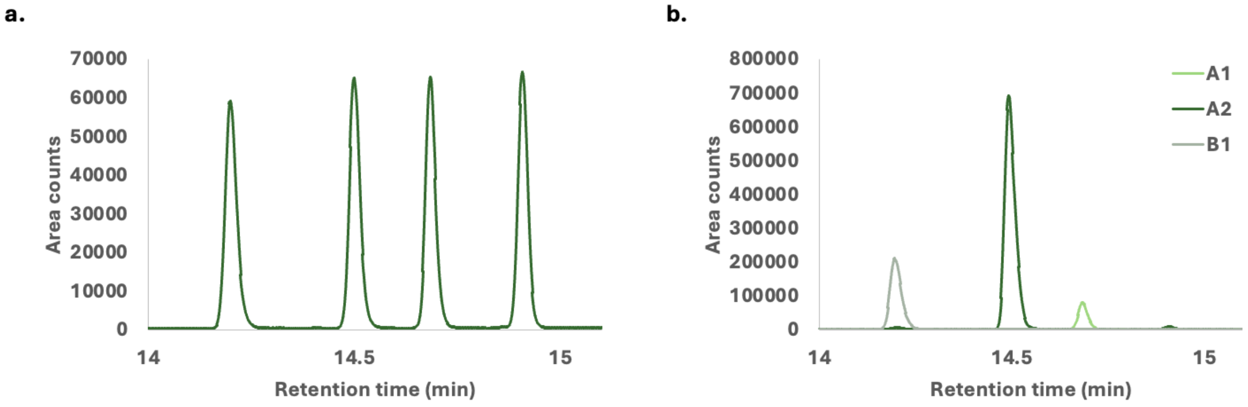

Figure S3: All four possible 3-hydroxyacid stereochemistry were measured on the GC-MS with a chiral column and verified via mass fragments. a) GC-MS separation of a racemic mixture of 3-hydroxy-2,4-dimethylpentanoic acid, giving the peaks in order of B1, A2, A1, and B2 respectively. b) Separation of the enantiomerically pure A1-, A2-, and B2-type products were run.

	Average RT	**EXPECTED**	**MEASURED**	**Secondary product**
A1	14.684	A1	A1	
A2	14.492	A2	A2	
B1	14.194	B1	B1	
Racemic peak 1	14.2	B1	B1	
Racemic peak 2	14.5	A2	A2	
Racemic peak 3	14.684	A1	A1	
Racemic peak 4	14.908	B2	B2	
mCherry (neg ctrl)	None	N/A	N/A	
Lip1-TE	14.496	A2	A2	
KR*		N/A	N/A	
Acu AT-KR-ACP	14.678	A1	A1	
Acu KR-ACP	14.678	A1	A1	
Acu AT-KR	14.674	A1	A1	
Acu KR	14.674	A1	A1	
Baf AT-KR-ACP	14.188	B1	B1	
Baf KR-ACP	14.184	B1	B1	
Baf AT-KR	14.182	B1	B1	
Baf KR	14.176	B1	B1	
Debs AT-KR-ACP	N/A	B2	N/A	
Debs KR-ACP	14.882	B2	B2	
Debs AT-KR	N/A	B2	N/A	
Debs KR	14.836	B2	B2	

AmpM1	N/A	A2	N/A	
PM1M1	14.42	A2	A2	
FD8M2	14.41	A2	A2	
StrM4	14.414	A2	A2	
LasM5	14.596	A2	A1	A2
BafM9	14.408	A2	A2	
SalM14	14.598	A2	A1	A2
TylM1	14.094	B1	B1	
AveM1	14.092	B1	B1	
ChaM1	14.092	B1	B1	
MeiM1	14.09	B1	B1	
AldM2	14.088	B1	B1	
NemM2	14.088	B1	B1	
PM1M4	14.086	B1	B1	
HerM6	14.084	B1	B1	
AcuM8	14.082	B1	B1	
NemM12	N/A	B1	N/A	
AcuM15	N/A	B1	N/A	
MegM1	N/A	B2	N/A	
LanM1	14.784	B2	B2	
FK5M2	14.782	B2	B2	
AntM2	14.776	B2	B2	
AcuM5	14.78	B2	B2	
HalM7	14.67	B2	N/A	
MerM10	14.778	B2	B2	
TylM1-DE	14.094	B1	B1	
ChaM1-DE	14.066	B1	B1	
BafM1-DE	14.058	B1	B1	
PM1M4-DE	14.054	B1	B1	

Table S2: The measured stereochemistry of each Lip1-TE variant is shown with the expected and measured stereochemistry, based on the GC-MS measurements. The retention times in the second column are separated as shown in **Figure S1**. N/A means the signal was too low or not present.

Chemical and sequence similarity analysis of KR domains

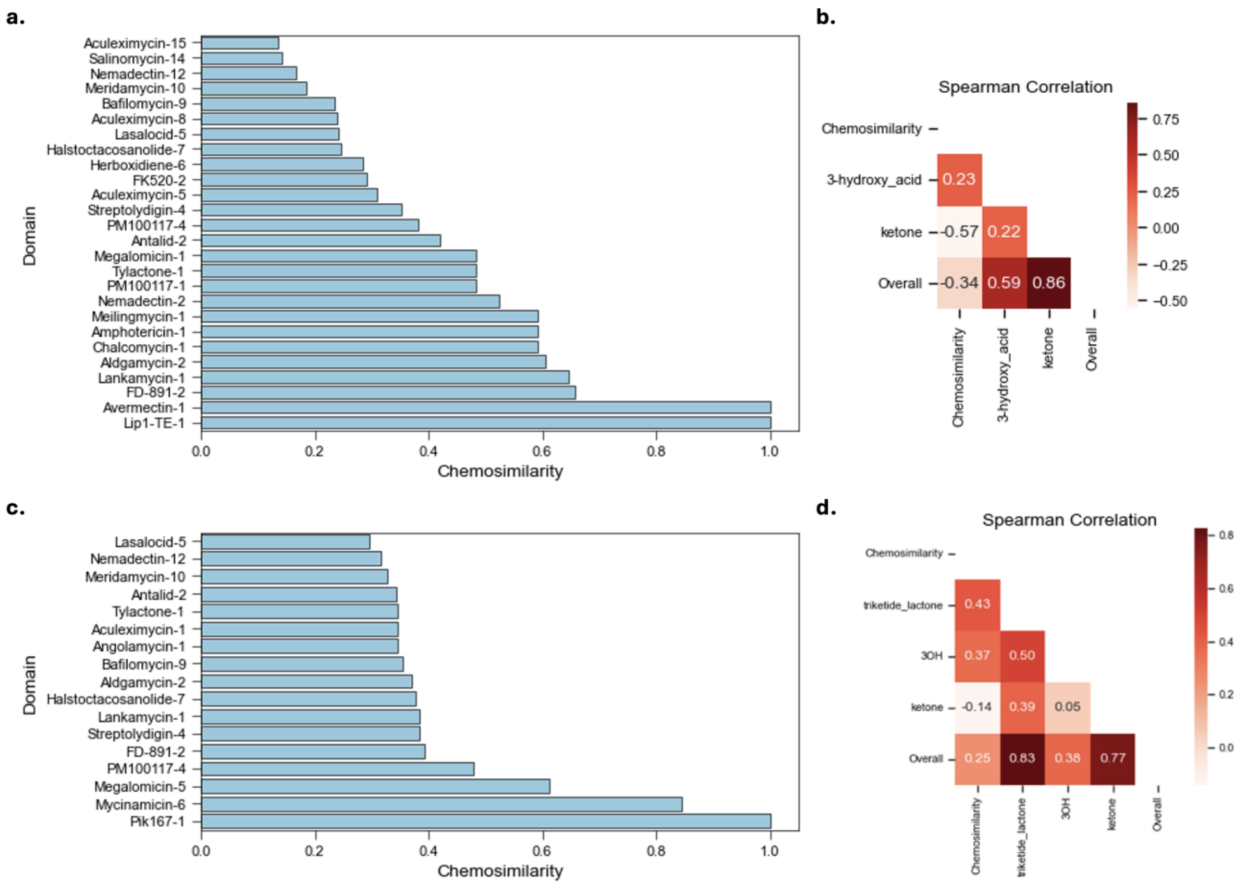

Figure S4: The chemical similarity of the KR donor domains used in this study were compared to the acceptor PKS substrates, Lip1-TE and Pik167, and evaluated against production data. a) The chemical similarity of the donor KR domains relative to the Lip1-TE KR. b) Spearman correlation coefficient relating, chemosimilarity, 3-hydroxyacid production, ketone production, and overall production (3-hydroxy acids + ketones) for Lip1-TE to one another are shown. c) The chemical similarity of the donor KR domains are shown relative to the Pik167 KR domain. d) Spearman correlation coefficients are shown for production in the KR domain exchanged Pik167 PKS, including triketide lactone production, side production of 3-hydroxyacids and ketones, along with overall production combining the three.

Proteomics measurements for Lip1-TE

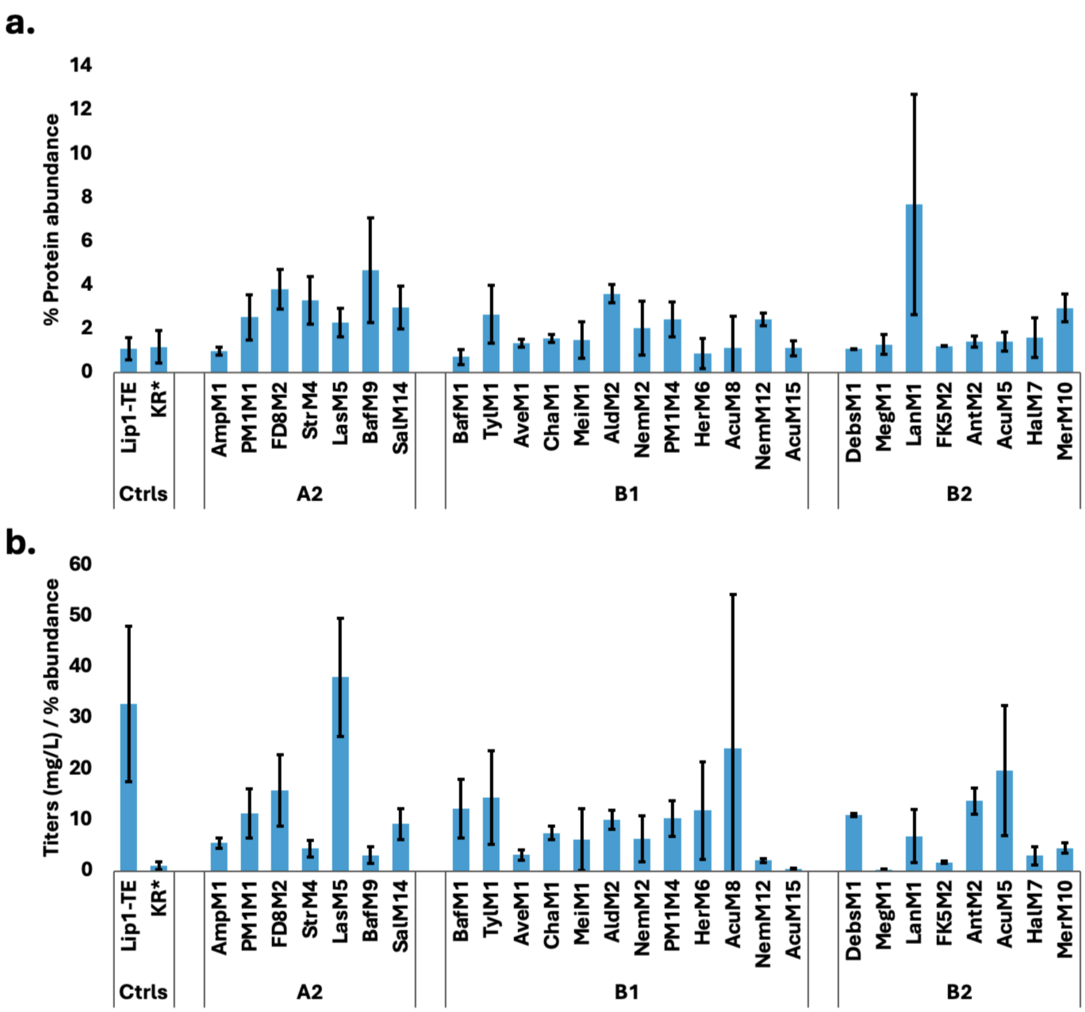

Figure S5: Proteomics measurements for the Lip1-TE KR domain exchanges in *S. albus* J1-74 are shown as % abundance of the targeted protein. a) The % abundance of all KR domain exchanges in Lip1-TE are shown. b) The titers from Lip1-TE KR domain exchanges are shown normalized to the % protein abundance

KR inactivation mutation for ketone production

```
Alb188|Alb188        ELLDHDSLDAFVLFSSIAGVWGSGDHGAFAAANAFLNALAEYNRARGIPTTSIAWGVWNA        180
Lips1TE|Lips1TE      ELLDHDSLDAFVLFSSIAGVWGSGDHGAYAAANAFLNALAEYNRARGIPTTSIAWGVWNA        180
Pik127|Pik127        ELTRDLDLDAFVLFSSVSSTLGIPGQGNYAPHNAYLDALAARRRATGRSAVSVAWGPWDG        179
Pik167|Pik167        DLLRGTPLDAFVLYSSNAGVWGSGSQGVYAAANAHLDALAARRRARGETATSVAWGLWAG        179
                     :*     ******:** :.. *  .:*|:*  **.*:***  .** *  :.*:*** * .
```

Figure S6: Alb188 is the KR inactivated version of Lip1-TE with a single point mutation of a tyrosine to a phenylalanine, made by Yuzawa et al.[5] The mutation was mapped onto Pik127 and Pik167 to abolish triketide lactone production and produce only ketones. Alignments were done using Clustal Omega within the EMBL-EBI analysis tools framework.[6,7]

Chiral column measurements for Pik127 and Pik167 on LC-MS

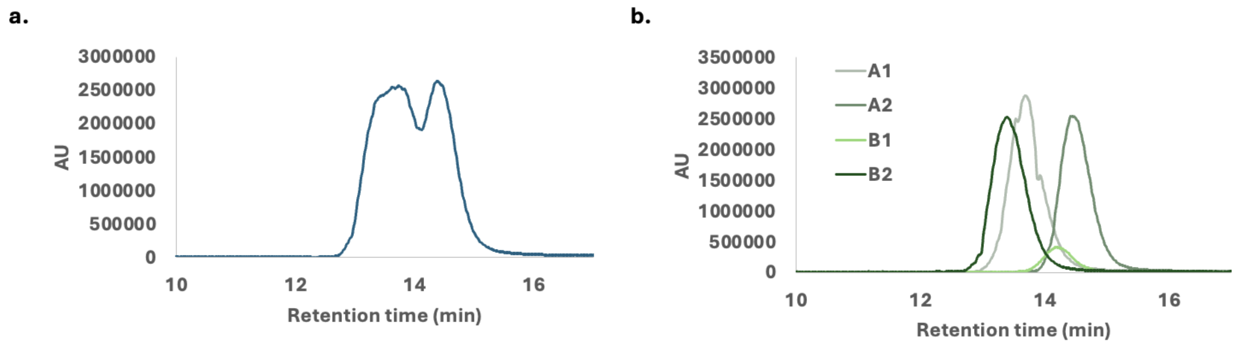

Figure S7: The separation of the triketide lactone product produced by Pik127 and Pik167 was done with LC-MS. All products were also observed at 247 nm. a) The racemic mixture of all four stereochemical configurations were extracted at 171 *m/z* in positive ion mode. b) All four enantiomerically pure triketide lactone standards are overlaid after being extracted at 171 *m/z* in positive ion mode.

Table S3: The measured stereochemistry of each Pik167 variant is shown here, where the second column is the averaged retention time based on the chiral LC-MS measurements and chromatograms shown in **Figure S6**. N/A means the signal was too low or not present.

	Average RT	**EXPECTED**	**MEASURED**
B2	13.2676667	B2	B2
B1	14.306	B1	B1
A2	14.2953333	A2	A2
A1	13.6496667	A1	A1
Pik167	13.5202333	A1	A1
AcuM1	13.6023333	A1	A1
AngM1	13.482	A1	A1
MegM5	13.551	A1	A1
MycM6	13.5826667	A1	A1
FD8M2	14.1653333	A2	A2
StrM4	13.77	A2	A1
LasM5	14.368	A2	A2
BafM9	13.6696667	A2	A1
TylM1	14.282	B1	B1
AldM2	14.2313333	B1	B1
PM1M4	14.325	B1	B1
NemM12	N/A	B1	N/A
LanM1	13.2523333	B2	B2
AntM2	14.3746667	B2	B1
HalM7	N/A	B2	N/A

MerM10	14.46	B2	B1
Pik167WT		A1	A1
Acu-Tri		A1	A1
Acu-Di		A1	A1
Acu-KR		A1	A1
Acu-KS		A1	N/A
Acu-Tri-KS		A1	N/A
Acu-Di-KS		A1	A1
Acu-KR-KS		A1	N/A
FD-Tri		A2	A2
FD-Di		A2	A2
FD-KR		A2	A2
FD-KS		A1	A1
FD-Tri-KS		A2	A2
FD-Di-KS		A2	A2
FD-KR-KS		A2	N/A
Tyl-Tri		B1	B1
Tyl-Di		B1	B1
Tyl-KR		B1	B1
Tyl-KS		A1	A1
Tyl-Tri-KS		B1	B1
Tyl-Di-KS		B1	N/A
Tyl-KR-KS		B1	B1
Lank-Tri		B2	B2
Lank-Di		B2	B2
Lank-KR		B2	B2
Lank-KS		A1	A1
Lank-Tri-KS		B2	B2
Lank-Di-KS		B2	B2
Lank-KR-KS		B2	B2

KS mutation activity with all KR domain exchanges

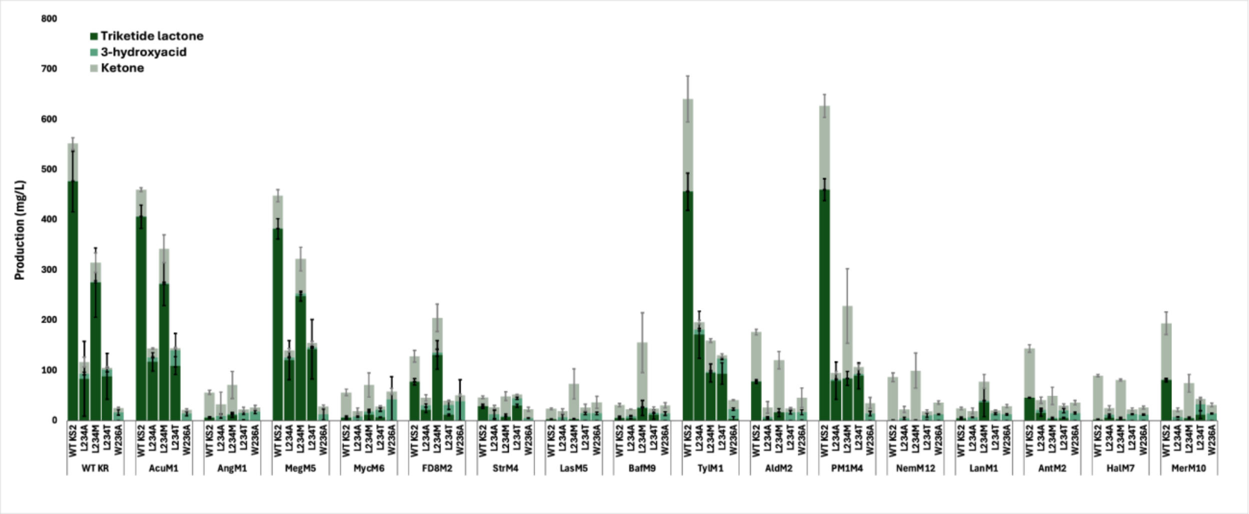

Figure S8: Each KR domain exchange in Pik167 was paired with the mutated version of the downstream KS, with either the L234A, L234M, L234T, or W236A variant.

Proteomics measurements for Pik127 and Pik167

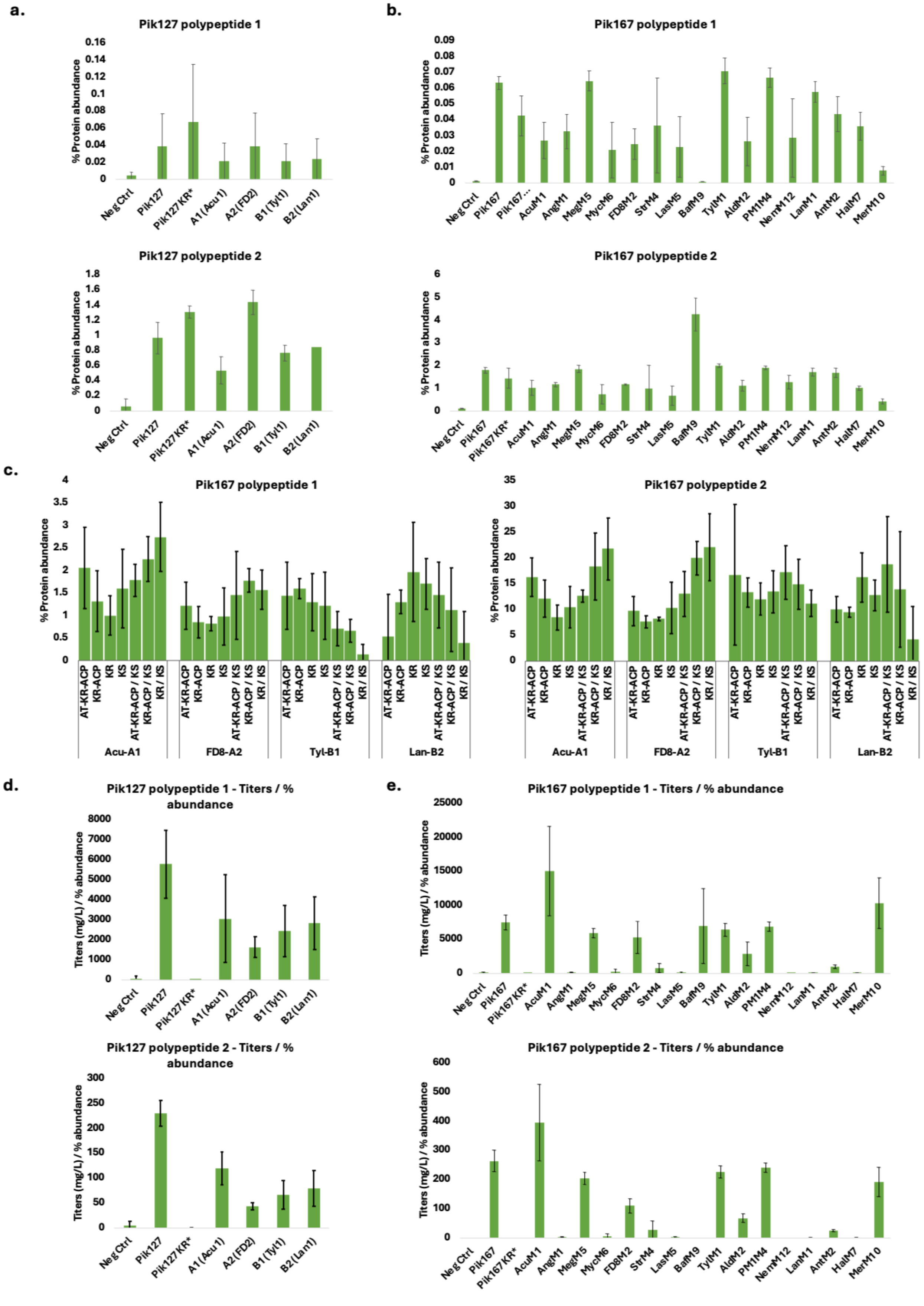
a.
Pik127 polypeptide 1
% Protein abundance
Neg Ctrl
Pik127
Pik127 KR*
A1 (Acu1)
A2 (FD2)
B1 (Tyl1)
B2 (Lan1)
Pik127 polypeptide 2
b.
Pik167 polypeptide 1
Neg Ctrl
Pik167
Pik167…
AcuM1
AngM1
MegM5
MycM6
FD8M2
StrM4
LasM5
BafM9
TylM1
AldM2
PM1M4
NemM12
LanM1
AntM2
HalM7
MerM10
Pik167 polypeptide 2
Pik167 KR*
c.
Pik167 polypeptide 1
Pik167 polypeptide 2
AT-KR-ACP
KR-ACP
KR
KS
AT-KR-ACP / KS
KR-ACP / KS
KR / KS
Acu-A1
FD8-A2
Tyl-B1
Lan-B2
d.
Pik127 polypeptide 1 - Titers / % abundance
Titers (mg/L) / % abundance
Pik127 polypeptide 2 - Titers / % abundance
e.
Pik167 polypeptide 1 - Titers / % abundance
Pik167 polypeptide 2 - Titers / % abundance

Figure S9: Proteomics measurements for all Pik127 and Pik167 experiments in K207-3 are shown as % abundance of the targeted protein. a) The % abundances of the first and second polypeptides in Pik127 are shown. b) The % abundances of the first and second polypeptide of KR domain exchanges Pik167 are shown. c) The % abundance of the first and second polypeptides in Pik167 for KS functional unit domain exchanges with the KR domain are shown. In all cases, error bars are the standard deviation of triplicates. d) The titers normalized to the protein % abundance are shown for all Pik127 KR domain exchanges. e) The titers for the KR domain exchanges in Pik167 are shown normalized to % abundance.

KS mutations in Pik167

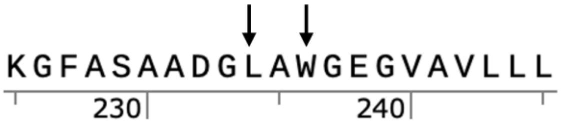

Figure S10: Amino acids L234 and W236 of the second KS domain in Pik167, KS module 5, were mutated.

Comparing production media for Pik127 and Pik167

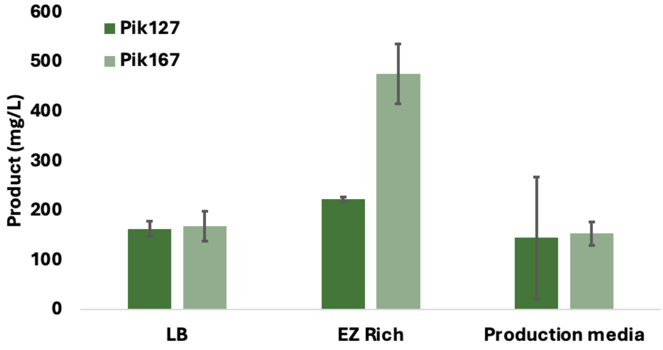

Figure S11: Triketide lactone production of Pik127 (K207-3 with ptm2 and ptm3) and Pik167 (K207-3 with ptm4 and ptm5) in LB, EZ Rich, and PKS production media derived from Miyazawa et al.[8]

Synthesis of triketide lactones - NMR spectra

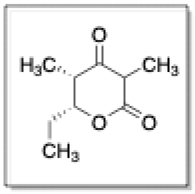

Lactone A

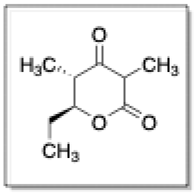

Lactone B

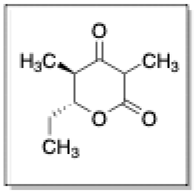

Lactone C

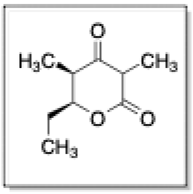

Lactone D

Four triketide lactone standards were prepared by synthetic organic chemistry. Detailed synthetic work about preparation and characterization of lactone A (compound S3), B (compound S6), C (compound S9), and D (compound S12) is shown below.

Unless stated otherwise, all chemicals, salts, and solvents were obtained from commercial suppliers (e.g. Sigma-Aldrich, VWR, Fisher Scientific) and used without further purification. All air- and moisture-sensitive manipulations were conducted using standard Schlenk techniques under an atmosphere of

nitrogen. Solvents and solutions were transferred using air-tight syringes. All flame-dried vessels were placed under vacuum and externally heated using a propane flame. All reactions were performed in flame-dried glassware under an atmosphere of nitrogen and were stirred using Teflon-coated magnetic stirring bars unless otherwise stated. Reactions were monitored by thin layer chromatography (TLC) on Baker-flex® precoated flexible plastic sheets and visualized by UV irradiation or stained by $KMnO_4$ TLC stain. Flash column chromatography was conducted with a Teledyne Isco CombiFlash NextGen 300 System using RediSep Gold Normal Phase Columns packed with 20-40 µm fine spherical silica gel.

Nuclear magnetic resonance (NMR) spectra were acquired on a Bruker AV600 at 600 MHz for ^{1}H and 125 MHz for ^{13}C at the NMR facility of the College of Chemistry, University of California, Berkeley. Chemical shifts were reported in ppm downfield of TMS and were referenced to residual solvent signal (^{1}H-NMR: $CDCl_3$ □ = 7.76 ppm, $CD_3C(O)OD$ = 2.03 ppm, CD_3OD = 3.31 ppm; ^{13}C-NMR: $CDCl_3$ □ = 77.16 ppm, $CD_3C(O)OD$ = 20.00 ppm, CD_3OD = 49.00 ppm). NMR Spectra are reported as follows: chemical shift (multiplicity, number of nuclei, coupling constants where applicable). Splitting is reported with the following symbols: s = singlet, d = doublet, t = triplet, q = quartet, dd = doublet of doublets, dt = doublet of triplets, td = triplet of doublets, m = multiplet. Coupling constants are reported in Hz.

Synthesis of lactone A (S3, (5S,6R)-6-ethyl-3,5-dimethyldihydro-2H-pyran-2,4(3H)-dione)

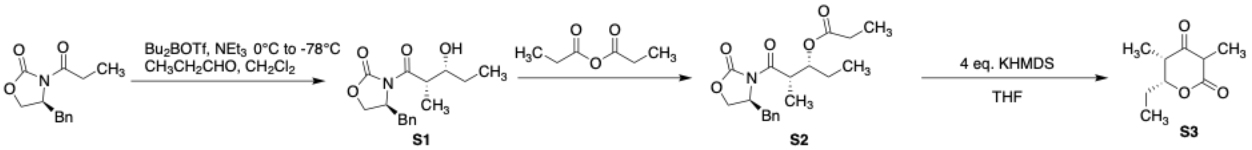

Compound **S1** was prepared as reported.[9] To a stirred solution of **S1** (568 mg, 1.95 mmol) in anhydrous dichloromethane (20 mL) under nitrogen was added propionic anhydride (324 µl, 2.54 mmol) followed by the addition of triethylamine (380 µl, 2.73 mmol) and DMAP (48 mg, 0.39 mmol). The resulting reaction mixture was heated by an oil bath (52 °C) to reflux for 3 hrs. After the reaction was cooled to room temperature, the reaction solution (DCM) was washed sequentially by 1N HCl (10 mL), sat. $NaHCO_3$ (10 mL) and brine (10 mL). The organic layer (DCM) was dried by anhydrous Na_2SO_4 and filtered. After evaporation of the solvent under reduced pressure, the residue was subjected to purification by column chromatography to give 589 mg of compound **S2** ((2S,3R)-1-((S)-4-benzyl-2-oxooxazolidin-3-yl)-2-methyl-1-oxopentan-3-yl propionate) in 87% yield.

Data of (2S,3R)-1-((S)-4-benzyl-2-oxooxazolidin-3-yl)-2-methyl-1-oxopentan-3-yl propionate (**S2**):
^{1}**H-NMR** (600 MHz, $CDCl_3$) □ (ppm) 7.34-7.30 (m, 2H), 7.29-7.25 (m, 1H), 7.22-7.18 (m, 2H), 5.16 (ddd, 1H, J = 8.4, 5.4, 3.6 Hz), 4.57-4.51 (m, 1H), 4.28 (t, 1H, J = 8.4 Hz), 4.15 (dd, 1H, J = 9.0, 1.8 Hz), 4.00 (ddd, 1H, J = 13.8, 7.2, 3.0 Hz), 3.27 (dd, 1H, J = 13.2, 3.0 Hz), 2.77 (dd, 1H, J = 13.2, 9.6 Hz), 2.32 (ddd, 2H, J = 15.0, 7.8, 3.0 Hz), 1.70-1.60 (m, 2H), 1.20 (d, 3H, J = 7.2 Hz), 1.13 (t, 3H, J = 7.8 Hz), 0.91(t, 3H, J = 7.2 Hz),
^{13}**C NMR** (151 MHz, $CDCl_3$) □ 174.42, 174.18, 153.73, 135.48, 129.44, 128.91, 127.28, 74.49, 66.38, 55.93, 41.12, 37.96, 27.64, 25.30, 10.07, 9.86, 9.32.

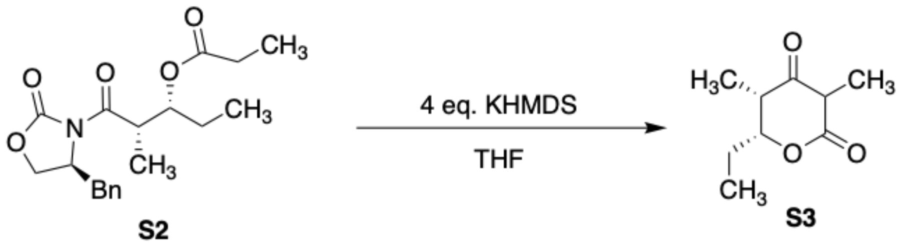

To a stirred solution of ((2S,3R)-1-((S)-4-benzyl-2-oxooxazolidin-3-yl)-2-methyl-1-oxopentan-3-yl propionate) (**S2**, 144 mg, 0.41 mmol) in anhydrous THF (3.4 mL) at -78 °C under nitrogen was added KHMDS (1.66 mL, 1.66 mmol, 1M solution in THF) and the resulting reaction mixture was continued to stir for 4h at this temperature. Then a mixture of sat. NH_4Cl:MeOH:H_2O = 10 mL:10 mL:10 mL was added to quench the reaction. After the cooling bath was removed, the reaction mixture was allowed to room temperature. EtOAc (10 mL) and water (3 mL) were added and two layers were separated in a separation funnel. The water layer was acidified with HCl (1N) to reach pH 2.0 (monitored by pH paper). Then extraction was performed by EtOAc (20 mL x 3). The combined organic phase was washed by brine and dried over anhydrous Na_2SO_4. After filtration and evaporation of the solvent under reduced pressure, the residue was purified by CombiFlash to give 30 mg lactone product **S3** in 43% yield.

Data of (5S,6R)-6-ethyl-3,5-dimethyldihydro-2H-pyran-2,4(3H)-dione **(Lactone A, S3)**:
^{1}**H-NMR** (600 MHz, $CDCl_3$) □ (ppm) 4.65 (ddd, 1H, J = 8.4, 5.4, 3.0 Hz), 3.62 (dd, 1H, J = 13.2, 6.6 Hz), 2.62 (ddd, 1H, J = 15.0, 7.8, 3.0 Hz), 1.90-1.82 (m, 1H), 1.68-1.62 (m, 1H), 1.35 (d, 3H, J = 6.6 Hz), 1.11 (d, 3H, J = 7.5 Hz), 1.06 (t, 3H, J = 7.4 Hz).
^{13}**C NMR** (151 MHz, $CDCl_3$) □ 205.48, 170.15, 78.55, 50.40, 44.37, 24.03, 9.90, 9.71, 8.23.

Synthesis of lactone B (S6, (5S,6S)-6-ethyl-3,5-dimethyldihydro-2H-pyran-2,4(3H)-dione)

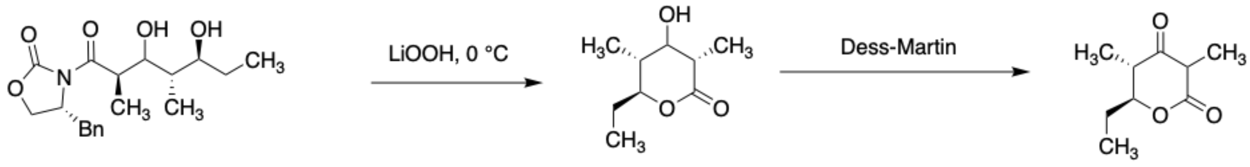

S4 **S5** **S6**

Compound **S4** was prepared according to the literature.[10] To a stirred solution of **S4** (35 mg, 0.10 mmol) in a mixture solvent (THF : H_2O = 2 mL : 0.5 mL) at 0 °C was added H_2O_2 (40 µL, 0.51 mmol) followed by the addition of LiOH (150 µL, 1M aqueous solution). The resulting mixture was stirred at 0 °C for 2 h and quenched by the dropwise addition of Na_2SO_3 (300 µL, 2 M aqueous solution). The resulting mixture was stirred at 0 °C for an additional 1 h. After evaporation of the volatile solvents, the resulting residue was extracted with dichloromethane (3 mL) to remove the oxazolidinone auxiliary. The organic phase was discarded, and the aqueous phase was acidified with 1M HCl to pH 3 and the resulting mixture was stirred at room temperature for 5 h. Then, the aqueous solution was extracted with ethyl acetate (15 mL X 3). The combined organic extracts were washed by brine and dried by anhydrous Na_2SO_4. After filtration and evaporation of the solvent under reduced pressure, the residue was purified by CombiFlash to give 10 mg lactone product **S5** in 59% yield.
Data of (3S,5R,6S)-6-ethyl-4-hydroxy-3,5-dimethyltetrahydro-2H-pyran-2-one **(S5)**:
^{1}**H-NMR** (600 MHz, $CDCl_3$) □ (ppm) 3.88 (ddd, 1H, J = 10.2, 6.6, 3.0 Hz), 3.31 (m, 1H), 2.39 (m, 1H), 1.94 (d, 1H, J = 5.4 Hz), 1.91-1.85 (m, 1H), 1.78-1.71 (m, 1H), 1.66-1.59 (m, 1H), 1.41 (d, 3H, J = 7.2 Hz), 1.09 (d, 3H, J = 6.6 Hz), 1.02 (t, 3H, J = 7.8 Hz).
^{13}**C NMR** (151 MHz, $CDCl_3$) □ 173.27, 82.75, 75.32, 44.49, 39.91, 25.76, 13.84, 13.15, 8.49.

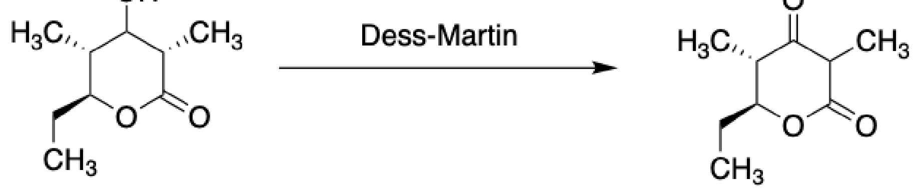

S5 **S6**

To a stirred solution of S5 (10 mg, 0.058 mmol) in dichloromethane (1.5 mL) was added Dess-Martin Periodinane (52 mg, 0.122 mmol). The resulting mixture was stirred at ambient temperature for 110 min, and then was purified by CombiFlash to give 4.5 mg of lactone **S6** in 45% yield.

Data of (5S,6S)-6-ethyl-3,5-dimethyldihydro-2H-pyran-2,4(3H)-dione` **(S6, lactone B)**:
^{1}**H-NMR** (600 MHz, $CDCl_3$) □ (ppm) 4.34 (ddd, 1H, J = 10.8, 7.8, 3.0 Hz), 3.53 (dd, 1H, J = 13.2, 6.6 Hz), 2.37-2.31 (m, 1H), 1.99-1.92 (m, 1H), 1.74-1.66 (m, 1H), 1.37 (d, 3H, J = 6.6 Hz), 1.20 (d, 3H, J = 7.2 Hz), 1.11 (t, 3H, J = 7.2 Hz).
^{13}**C NMR** (151 MHz, $CDCl_3$) □ 204.72, 169.91, 80.27, 50.17, 45.97, 25.10, 12.06, 8.74, 8.05.

Synthesis of lactone (S9, lactone C, (5R,6R)-6-ethyl-3,5-dimethyldihydro-2H-pyran-2,4(3H)-dione)

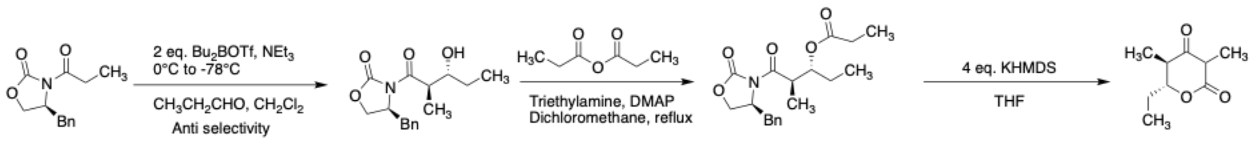

S7 S8 S9

Compound **S7** was prepared as reported.[11] To a stirred solution of **S7** (392 mg, 1.35 mmol) in anhydrous dichloromethane (20 mL) under nitrogen was added propionic anhydride (223 µl, 1.75 mmol) followed by the addition of triethylamine (263 µl, 1.89 mmol) and DMAP (33 mg, 0.27 mmol). The resulting reaction mixture was heated by an oil bath (52 °C) to reflux for 3 hrs. After the reaction was cooled to room temperature, the reaction solution (DCM) was washed sequentially by 1N HCl (10 mL), sat. $NaHCO_3$ (10 mL) and brine (10 mL). The organic layer (DCM) was dried by anhydrous Na_2SO_4 and filtered. After evaporation of the solvent under reduced pressure, the residue was subjected to purification by column chromatography to give 360 mg of compound **S8** ((5R,6R)-6-ethyl-3,5-dimethyldihydro-2H-pyran-2,4(3H)-dione) in 77% yield.

Data of (5R,6R)-6-ethyl-3,5-dimethyldihydro-2H-pyran-2,4(3H)-dione (**S8**):
^{1}**H-NMR** (600 MHz, $CDCl_3$) □ (ppm) 7.36-7.32 (m, 2H), 7.30-7.26 (m, 1H), 7.24 (m, 2H), 5.26 (ddd, 1H, J = 10.8, 7.2, 3.6 Hz), 4.70-4.65 (m, 1H), 4.20-4.12 (m, 3H), 3.27 (dd, 1H, J = 13.2, 3.6 Hz), 2.69 (dd, 1H, J = 13.2, 9.6 Hz), 2.35-2.23 (m, 2H), 1.88-1.82 (m, 1H), 1.64-1.56 (m, 1H), 1.19 (d, 3H, J = 7.2 Hz), 1.12 (t, 3H, J = 7.8 Hz), 0.93(t, 3H, J = 7.2 Hz),
^{13}**C NMR** (151 MHz, $CDCl_3$) □ 174.58, 173.53, 153.04, 135.20, 127.40, 75.41, 65.86, 55.29, 53.46, 40.76, 37.84, 27.70, 24.05, 14.02, 9.27, 8.77.

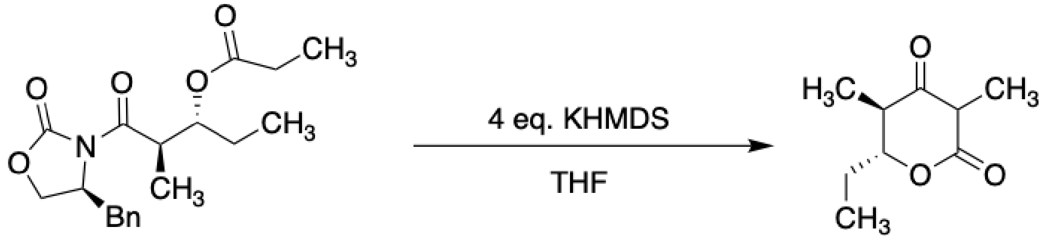

S8 S9

To a stirred solution of ((5R,6R)-6-ethyl-3,5-dimethyldihydro-2H-pyran-2,4(3H)-dione) (**S8**, 148 mg, 0.43 mmol) in anhydrous THF (3.5 mL) at -78 °C under nitrogen was added KHMDS (1.71 mL, 1.71 mmol, 1M solution in THF) and the resulting reaction mixture was continued to stir for 4h at this temperature. Then a mixture of sat. NH_4Cl:MeOH:H_2O = 10 mL:10 mL:10 mL was added to quench the reaction. After the cooling bath was removed, the reaction mixture was allowed to room temperature. EtOAc (10 mL) and water (3 mL) were added, and two layers were separated in a separation funnel. The water layer was acidified with HCl (1N) to reach pH 2.0 (monitored by pH paper). Then extraction was performed by EtOAc

(20 mL x 3). The combined organic phase was washed by brine and dried over anhydrous Na_2SO_4. After filtration and evaporation of the solvent under reduced pressure, the residue was purified by CombiFlash to give 54 mg lactone product **S9** in 74% yield.

Data of (5R,6R)-6-ethyl-3,5-dimethyldihydro-2H-pyran-2,4(3H)-dione **(Lactone C, S9)**:
^{1}**H-NMR** (600 MHz, $CDCl_3$) □ (ppm) 4.34 (ddd, 1H, J = 10.8, 7.8, 3.0 Hz), 3.53 (dd, 1H, J = 13.2, 6.6 Hz), 2.37-2.30 (m, 1H), 1.98-1.91 (m, 1H), 1.74-1.65 (m, 1H), 1.36 (d, 3H, J = 6.6 Hz), 1.20 (d, 3H, J = 7.2 Hz), 1.10 (t, 3H, J = 7.2 Hz).
^{13}**C NMR** (151 MHz, $CDCl_3$) □ 204.73, 169.33, 80.77, 50.17, 45.96, 25.09, 12.05, 8.73, 8.04.

Synthesis of lactone D (S12, (5R,6S)-6-ethyl-3,5-dimethyldihydro-2H-pyran-2,4(3H)-dione)

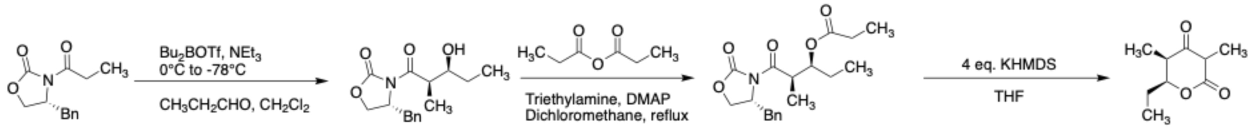

S10 **S11** **S12**

Compound **S10** was prepared as reported [4].[12] To a stirred solution of **S10** (404 mg, 1.39 mmol) in anhydrous dichloromethane (20 mL) under nitrogen was added propionic anhydride (230 µl, 1.80 mmol) followed by the addition of triethylamine (271 µl, 1.95 mmol) and DMAP (34 mg, 0.28 mmol). The resulting reaction mixture was heated by an oil bath (52 °C) to reflux for 3 hrs. After the reaction was cooled to room temperature, the reaction solution (DCM) was washed sequentially by 1N HCl (10 mL), sat. $NaHCO_3$ (10 mL) and brine (10 mL). The organic layer (DCM) was dried by anhydrous Na_2SO_4 and filtered. After evaporation of the solvent under reduced pressure, the residue was subjected to purification by column chromatography to give 390 mg of compound **S11** ((2R,3S)-1-((R)-4-benzyl-2-oxooxazolidin-3-yl)-2-methyl-1-oxopentan-3-yl propionate) in 81% yield.

Data of (2R,3S)-1-((R)-4-benzyl-2-oxooxazolidin-3-yl)-2-methyl-1-oxopentan-3-yl propionate (**S11**):
^{1}**H-NMR** (600 MHz, $CDCl_3$) □ (ppm) 7.34-7.30 (m, 2H), 7.28-7.25 (m, 1H), 7.22-7.18 (m, 2H), 5.16 (ddd, 1H, J = 9.0, 5.4, 3.6 Hz), 4.56-4.51 (m, 1H), 4.28 (dd, 1H, J = 9.0, 7.8 Hz), 4.15 (dd, 1H, J = 9.0, 2.4 Hz), 4.00 (ddd, 1H, J = 13.8, 6.6, 3.6 Hz), 3.27 (dd, 1H, J = 13.2, 3.6 Hz), 2.76 (dd, 1H, J = 13.2, 9.6 Hz), 2.32 (ddd, 2H, J = 15.6, 7.8, 2.4 Hz), 1.69-1.61 (m, 2H), 1.20 (d, 3H, J = 7.2 Hz), 1.14 (t, 3H, J = 7.8 Hz), 0.91(t, 3H, J = 7.2 Hz),
^{13}**C NMR** (151 MHz, $CDCl_3$) □ 174.42, 174.18, 153.73, 135.48, 129.44, 128.91, 127.28, 74.49, 66.38, 55.93, 41.12, 37.97, 27.65, 25.30, 10.07, 9.86, 9.32.

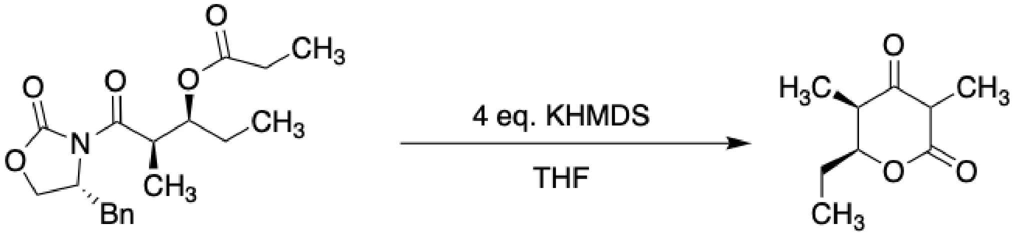

S11 **S12**

To a stirred solution of (2R,3S)-1-((R)-4-benzyl-2-oxooxazolidin-3-yl)-2-methyl-1-oxopentan-3-yl propionate (**S11**, 154 mg, 0.44 mmol) in anhydrous THF (3.5 mL) at -78 °C under nitrogen was added KHMDS (1.78 mL, 1.78 mmol, 1M solution in THF) and the resulting reaction mixture was continued to stir for 4h at this temperature. Then a mixture of sat. NH_4Cl:MeOH:H_2O = 10 mL:10 mL:10 mL was added to quench the reaction. After the cooling bath was removed, the reaction mixture was allowed to room

temperature. EtOAc (10 mL) and water (3 mL) were added and two layers were separated in a separation funnel. The water layer was acidified with HCl (1N) to reach pH 2.0 (monitored by pH paper). Then extraction was performed by EtOAc (20 mL x 3). The combined organic phase was washed by brine and dried over anhydrous Na_2SO_4. After filtration and evaporation of the solvent under reduced pressure, the residue was purified by CombiFlash to give 33 mg lactone product **S12** in 44% yield.

Data of (5R,6S)-6-ethyl-3,5-dimethyldihydro-2H-pyran-2,4(3H)-dione **(Lactone D, S12)**:
^{1}**H-NMR** (600 MHz, $CDCl_3$) □ (ppm) 4.65 (ddd, 1H, J = 8.4, 5.4, 3.0 Hz), 3.61 (dd, 1H, J = 13.2, 7.2 Hz), 2.62 (ddd, 1H, J = 15.0, 7.8, 3.0 Hz), 1.90-1.82 (m, 1H), 1.68-1.62 (m, 1H), 1.35 (d, 3H, J = 6.6 Hz), 1.11 (d, 3H, J = 7.5 Hz), 1.07 (t, 3H, J = 7.4 Hz).
^{13}**C NMR** (151 MHz, $CDCl_3$) □ 205.43, 170.11, 78.55, 50.40, 44.38, 24.04, 9.91, 9.71, 8.24.

(1) Yuzawa, S.; Deng, K.; Wang, G.; Baidoo, E. E. K.; Northen, T. R.; Adams, P. D.; Katz, L.; Keasling, J. D. Comprehensive in Vitro Analysis of Acyltransferase Domain Exchanges in Modular Polyketide Synthases and Its Application for Short-Chain Ketone Production. *ACS Synth. Biol.* **2017**, *6* (1), 139–147. https://doi.org/10.1021/acssynbio.6b00176.
(2) Eng, C. H.; Yuzawa, S.; Wang, G.; Baidoo, E. E. K.; Katz, L.; Keasling, J. D. Alteration of Polyketide Stereochemistry from *Anti* to *Syn* by a Ketoreductase Domain Exchange in a Type I Modular Polyketide Synthase Subunit. *Biochemistry* **2016**, *55* (12), 1677–1680. https://doi.org/10.1021/acs.biochem.6b00129.
(3) Massicard, J.-M.; Soligot, C.; Weissman, K. J.; Jacob, C. Manipulating Polyketide Stereochemistry by Exchange of Polyketide Synthase Modules. *Chem. Commun.* **2020**, *56* (84), 12749–12752. https://doi.org/10.1039/D0CC05068G.
(4) Keatinge-Clay, A. T. The Structures of Type I Polyketide Synthases. *Nat. Prod. Rep.* **2012**, *29* (10), 1050–1073. https://doi.org/10.1039/C2NP20019H.
(5) Yuzawa, S.; Mirsiaghi, M.; Jocic, R.; Fujii, T.; Masson, F.; Benites, V. T.; Baidoo, E. E. K.; Sundstrom, E.; Tanjore, D.; Pray, T. R.; George, A.; Davis, R. W.; Gladden, J. M.; Simmons, B. A.; Katz, L.; Keasling, J. D. Short-Chain Ketone Production by Engineered Polyketide Synthases in Streptomyces Albus. *Nat. Commun.* **2018**, *9* (1), 4569. https://doi.org/10.1038/s41467-018-07040-0.
(6) Sievers, F.; Higgins, D. G. The Clustal Omega Multiple Alignment Package. In *Multiple Sequence Alignment: Methods and Protocols*; Katoh, K., Ed.; Springer US: New York, NY, 2021; pp 3–16. https://doi.org/10.1007/978-1-0716-1036-7_1.
(7) Madeira, F.; Madhusoodanan, N.; Lee, J.; Eusebi, A.; Niewielska, A.; Tivey, A. R. N.; Lopez, R.; Butcher, S. The EMBL-EBI Job Dispatcher Sequence Analysis Tools Framework in 2024. *Nucleic Acids Res.* **2024**, *52* (W1), W521–W525. https://doi.org/10.1093/nar/gkae241.
(8) Miyazawa, T.; Fitzgerald, B. J.; Keatinge-Clay, A. T. Preparative Production of an Enantiomeric Pair by Engineered Polyketide Synthases. *Chem. Commun.* **2021**, *57* (70), 8762–8765. https://doi.org/10.1039/D1CC03073F.
(9) Castonguay, R.; He, W.; Chen, A. Y.; Khosla, C.; Cane, D. E. Stereospecificity of Ketoreductase Domains of the 6-Deoxyerythronolide B Synthase. *J. Am. Chem. Soc.* **2007**, *129* (44), 13758–13769. https://doi.org/10.1021/ja0753290.
(10) Zhao, Z.; Wu, Y.; Li, Y. On the Synthesis of Stereocalpin A: Partial Retraction/Correction of Previous Results and Rationalization of the Hidden Difficulties: Monitoring of Rutin in Human Urine by FI-CL. *Chin. J. Chem.* **2017**, *35*. https://doi.org/10.1002/cjoc.201600884.
(11) Willwacher, J.; Kausch-Busies, N.; Fürstner, A. Divergent Total Synthesis of the Antimitotic Agent Leiodermatolide. *Angew. Chem. Int. Ed.* **2012**, *51* (48), 12041–12046. https://doi.org/10.1002/anie.201206670.
(12) *Formal Total Synthesis of Kendomycin by Way of Alkyne Metathesis/Gold Catalysis - Hoffmeister - 2014 - Chemistry – A European Journal - Wiley Online Library*. https://chemistry-europe.onlinelibrary.wiley.com/doi/10.1002/chem.201304580 (accessed 2025-04-14).
